# Exploring the effects of dietary inulin in rainbow trout fed a high-starch, 100% plant-based diet

**DOI:** 10.1186/s40104-023-00951-z

**Published:** 2024-01-22

**Authors:** Raphaël Defaix, Jep Lokesh, Laura Frohn, Mickael Le Bechec, Thierry Pigot, Vincent Véron, Anne Surget, Sandra Biasutti, Frédéric Terrier, Sandrine Skiba-Cassy, Jérôme Roy, Stéphane Panserat, Karine Ricaud

**Affiliations:** 1 Université de Pau Et Des Pays de L’Adour, E2S UPPA, INRAE, NUMEA, Saint-Pée-Sur-Nivelle, France; 2 Université de Pau Et Des Pays de L’Adour, E2S UPPA, CNRS, IMT Mines Ales, IPREM, Pau, France; 3 Université de Pau Et Des Pays de L’Adour, E2S UPPA, IUT des Pays de l’Adour, Département Génie Biologique, Mont de Marsan, France

**Keywords:** Aquaculture, Fish nutrition, Gut microbiota, Immune markers, Intermediary metabolism, Inulin, Prebiotic, Rainbow trout, Short-chain fatty acids

## Abstract

**Background:**

High dietary carbohydrates can spare protein in rainbow trout (*Oncorhynchus mykiss*) but may affect growth and health. Inulin, a prebiotic, could have nutritional and metabolic effects, along with anti-inflammatory properties in teleosts, improving growth and welfare. We tested this hypothesis in rainbow trout by feeding them a 100% plant-based diet, which is a viable alternative to fishmeal and fish oil in aquaculture feeds. In a two-factor design, we examined the impact of inulin (2%) as well as the variation in the carbohydrates (CHO)/plant protein ratio on rainbow trout. We assessed the influence of these factors on zootechnical parameters, plasma metabolites, gut microbiota, production of short-chain fatty acids and lactic acid, as well as the expression of free-fatty acid receptor genes in the mid-intestine, intermediary liver metabolism, and immune markers in a 12-week feeding trial.

**Results:**

The use of 2% inulin did not significantly change the fish intestinal microbiota, but interestingly, the high CHO/protein ratio group showed a change in intestinal microbiota and in particular the beta diversity, with 21 bacterial genera affected, including *Ralstonia*, *Bacillus*, and 11 lactic-acid producing bacteria. There were higher levels of butyric, and valeric acid in groups fed with high CHO/protein diet but not with inulin. The high CHO/protein group showed a decrease in the expression of pro-inflammatory cytokines (*il1b, il8*, and *tnfa*) in liver and a lower expression of the genes coding for tight-junction proteins in mid-intestine (*tjp1a* and *tjp3*). However, the 2% inulin did not modify the expression of plasma immune markers. Finally, inulin induced a negative effect on rainbow trout growth performance irrespective of the dietary carbohydrates.

**Conclusions:**

With a 100% plant-based diet, inclusion of high levels of carbohydrates could be a promising way for fish nutrition in aquaculture through a protein sparing effect whereas the supplementation of 2% inulin does not appear to improve the use of CHO when combined with a 100% plant-based diet.

**Supplementary Information:**

The online version contains supplementary material available at 10.1186/s40104-023-00951-z.

## Background

The production of fish meal and fish oil for aquaculture requires more than 20 million tons of wild caught fish every year [1]. Beyond causing a disastrous environmental impact, this scale of industrial fishing leads to a decline of fish stocks in the oceans. Additionally, the price of fishmeal (FM) and fish oil (FO) are on a rise and expected to increase further until 2030 due to the growing aquaculture sector [1]. To overcome these sustainability issues, aquafeeds made from 100% plant-based ingredients are being researched increasingly, as an alternative to FM/FO [2–4]. In fact, this alternative allows ecological and economic benefits since the ingredients are cheaper, often more sustainable, and more available than marine ingredients. However, the 100% plant-based diet lead to a lower growth rates in carnivorous fish such as rainbow trout (*Oncorhynchus mykiss*) [5]. In a recent study, we replaced a part of the plant-proteins by 20% of digestible starch [6]. We showed for the first time that using digestible starch in rainbow trout in a 100% plant-based diet did not induce metabolic complications, i.e., no post-prandial hyperglycemia, and no glycogen and lipid accumulation in the liver, suggesting an efficient metabolic adaptation of rainbow trout to 20% of starch. In addition, we observed, a shift in the abundance of intestinal microbes such as the *Ralstonia* and *Bacillus*, accompanied by a higher production, in the digestive contents, of short-chain fatty acids (SCFAs) and lactate which are key molecules in the microbiota-host cross-talk [7]. Among the many critical factors in fish nutrition and feed efficiency, the intestinal microbiome plays an important role in several important functions such as energy production, nutrient metabolism, fermentation of dietary non-digestible components and immunity [8–10].

Prebiotics such as inulin, are known to modulate different metabolic and immune processes in mammals through direct or indirect actions on microbiota [11]. This non-digestible polysaccharide is fermented by intestinal microbes resulting in the production of various metabolites, mainly SCFAs [12, 13]. The SCFA molecules serve as an energy source for the intestinal cells [14], and induce to the activation of the free fatty acid receptors (*ffar*). SCFAs are also transported to the liver via the portal vein and are involved in different metabolic pathways: de novo lipogenesis, cholesterogenesis, fat storage [15, 16] as well as glucose homeostasis through regulation of gluconeogenic genes [17, 18]. Furthermore, SCFAs play an important role in regulating the integrity of the epithelial barrier of the intestinal in mucosa [19], and have anti-inflammatory functions [20, 21]. Inulin is extensively used in aquaculture for its beneficial effects. Inulin is known to improve the growth performance of fish [22–25] and known to be associated with a higher activity of intestinal digestive enzymes such as protease, amylase and lipase leading to a better availability of the nutrients for the host and for the microbiota [26, 27]. Additionally, the use of inulin is known to induce a shift in the diversity of microbes such as lactic-acid bacteria (*Lactobacillus*, *Weissella*) and *Bacillus* species [2, 28]. This modulation of microbiota through the use of prebiotics can induce the proliferation of beneficial intestinal bacteria that stimulate immune response and restrict the proliferation of pathogenic bacteria [23, 27, 29, 30].

It has been shown that the use of inulin in the diet reduces the metabolic disorder induced by a high carbohydrate (CHO) diet in Nile tilapia (*Oreochromis niloticus*) through modification of the expression of genes involved in metabolic and immune pathways [31], suggesting a better use of high level of dietary carbohydrates. The use of inulin in combination with a 100% plant-based diet (without carbohydrate supplementation) has also been tested in juvenile rainbow trout [2, 3]. These studies showed that the use of 2% inulin resulted in higher expression of several genes involved in different metabolic pathways (e.g. gluconeogenesis and glycolysis) and the higher expression of fatty acid receptor (*ffar*) genes in liver, indicating the possible interaction between the liver and the SCFA metabolites. Moreover, in the prebiotics-fed groups, differential abundances of *Lactobacillus* and *Bacillus* were observed which possibly indicates a “prebiotics-microbiome-host axis”. Together, these studies showed that inulin could be a promising ingredient to be incorporated in a high-CHO, 100% plant-based diet.

In this study, in a two-factorial design, we investigated the effect of dietary digestible starch (CHO/protein ratio) and inulin on the growth performance, host metabolism (gene expression in liver and intestine), intestinal inflammation and intestinal microbiome of rainbow trout fed a 100% plant-based diet. In addition, microbially derived metabolites such as SCFAs were measured as these are known to be key intermediary molecules between the gut microbiome and the host.

## Methods

### Ethical approval

The feeding experiment was conducted in accordance with the guidelines laid down by French and European legislation for the use and care of laboratory animals (Decree no. 2013–36, February 1^st^ 2013 and Directive 2010/63/EU, respectively). The fish handling protocols and the sampling procedures were described by the INRAE ethics committee (INRAE, 2002–36, April 14, 2002). The INRAE experimental station (Donzacq, Landes, France) is certified for animal experiments under the license number A40-228.1 by the French veterinary service, which is a competent authority.

### Diet and experimental setup

**Fig. 1:**
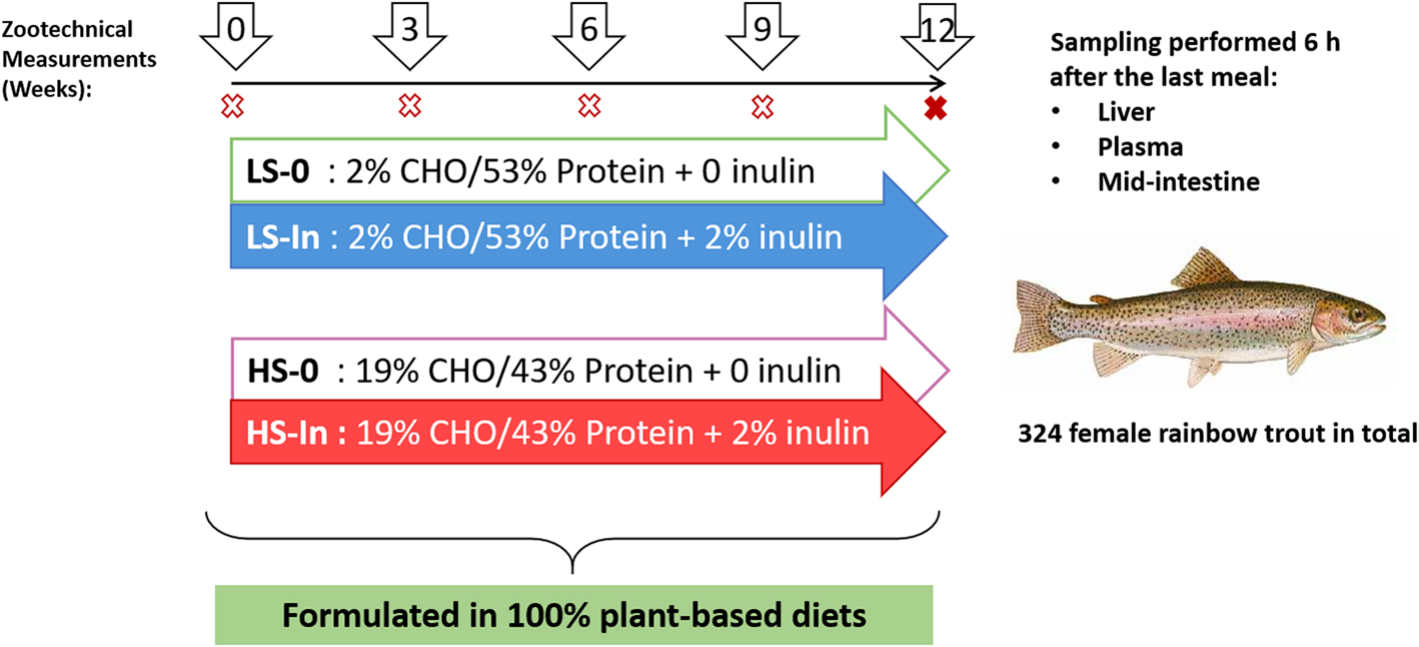
Experimental design of the feeding trial. Four experimental diets were produced as extruded pellets. These diets were made of 100% plant raw material and contained either 19% of digestible starch (high-starch diet) or 2% digestible starch (low-starch diet) and with 2% of inulin or 0 of inulin were produced as extruded pellets. The fish were fed the experimental diets by hand, twice a day, during 12 weeks to 324 females rainbow trout (~ 31 g) distributed in 12 tanks (3 tanks per group). The trout were weighed every 3 weeks to record the zootechnical parameters (*n* = 3 tank per experimental diet)

Four experimental 100% plant-based diets were formulated in this study. The high-starch (HS) diets contained a high carbohydrate to protein ratio, with 19% digestible CHO and 43% protein. The low-starch (LS) diet contained a low CHO to protein ratio, with 2% of digestible CHO and 53% protein. The diets with inulin were incorporated with 2% inulin while the diet without inulin had 2% cellulose supplementation, resulting in four experimental diets: LS-0, LS-In, HS-0, and HS-In (Table 1). These diets were isolipidic (21.43% ± 1.08% crude fat (DM baisis)) and isoenergetic (24.76 ± 0.39 kJ/g DM). The diets were extruded as pellets and were fed to fish manually twice a day (with an interval of 8 h) during 12 weeks. Three tanks (130 L) per group were used, each one containing 21 juvenile female rainbow trout (initial weight: 31.80 ± 0.11 g). During the experimental period, the fish were kept under standard rearing conditions with constant water temperature (17 °C, pH 7.5), constant water flow (0.3 L/s), and oxygen levels (9 mg/L). Fish mortality was checked (Additional file 1: Table S1) every day, and the tanks were weighed every 3 weeks to evaluate the growth and zootechnical parameters (Fig. 1).

**Table 1:** Formulation and proximate composition of four experimental diets

	**LS-0**	**LS-In**	**HS-0**	**HS-In**
**Ingredients, %**				
Wheat gluten	15	15	20	20
Gelatinized wheat starch	-	-	15	15
Whole wheat	-	-	4.86	4.86
Lysamine^®^ pea protein concentrate	16	16	20	20
Soybean meal	15	15	-	-
Soybean protein concentrate	12	12	-	-
Corn gluten	7.24	7.24	5.44	5.44
Faba protein concentrate	-	-	5	5
Lupin flour	4.53	4.53	-	-
Inulin	0	2	0	2
Cellulose	2	0	2	0
Rapeseed oil	10.74	10.74	11.91	11.91
Linseed oil	6	6	5.29	5.29
Palm oil	2	2	2	2
Dicalcium Phosphate	2	2	2	2
L-Lysine	1	1	0.5	0.5
L-Methionine	1	1	0.5	0.5
Soy lecithin powder	2.5	2.5	2.5	2.5
Premix Vitamins	1.5	1.5	1.5	1.5
Premix minerals	1.5	1.49	1.5	1.5
**Proximate composition**				
Dry matter (DM), %	96.98	96.17	96.32	95.69
Starch, % DM	2.57	2.41	19.22	19.32
Proteins, % DM	53.31	53.72	43.67	43.62
Lipids, % DM	23.06	22.49	22.74	23.15
Energy, kJ/g DM	25.14	25.10	24.40	24.39
Ash, % DM	5.86	5.98	4.99	5.00

### Sampling

At the end of the 12 weeks rearing, 12 fish per group (4 fish per tank) were sampled randomly, 6 h after the last meal. The fish were anaesthetized with benzocaine (50 mg/L) and euthanized by a benzocaine overdose (150 mg/L). Plasma, liver, and mid intestinal tissues were sampled from these fish and immediately frozen in liquid nitrogen. For microbiota analysis, the digestive-contents were recovered using sterile glass slide from the mid intestinal section which have been previously described as intestinal part involved in food digestion in rainbow trout [32]. In addition, the mid intestine and distal intestinal tissues were collected for RNA extraction. The digestive contents from two additional fish per tank were sampled for the SCFA measurement.

### Diets and whole-body composition

Dry matter of the diet and fish whole body was obtained by drying the samples at 105 °C for 24 h. The weight of the post-dried samples was subtracted from the pre-died samples. Ash content was measured by incinerating the samples at 550 °C for 16 h. Protein content was measured by the Kjeldahl™ method (FOSS, Denmark). Lipid content was determined by the Soxtherm method (Gerhardt analytical systems, Königswinter, Germany). Gross energy was measured with an adiabatic bomb calorimeter (IKA, Heitersheim Gribheimer, Germany). Starch contents was determined using the Megazyme^®^ (Bray, Wicklow, Ireland) total starch assay procedure.

### Quantification of plasma and liver metabolites

Blood was sampled for plasma collection from the caudal vein using heparinized syringes and tubes and then was centrifuged at 12,000 × *g* at 4 °C for 5 min. The plasma samples were stored at −20 °C until analyses. Commercial kits were used to determine the level of different plasma metabolites: glucose (W1306, Sobioda, Montbonnot-Saint-Martin, France), lactate (LC2389, kit Randox, Crumlin, United Kingdom), triglycerides (WTRIG1000, Sobioda), and cholesterol (W1W991, Sobioda). These kits were adapted to 96-well plate format according to the manufacturer’s instructions. Liver glycogen were measured using a protocol previously described by Good et al. [33].

### Microbial composition analysis

#### DNA extraction

The DNA extraction was performed on digestive contents from mid intestinal region (about 200 mg) using the QIAamp Fast DNA Stool Mini kit (51604, Qiagen, Hilden, Germany) according to the manufacturer’s instructions. The DNA quality was assessed for purity, and quantified with a microplate spectrophotometer (Epoch2, BioTek, France).

#### Generation of the (V3–V4) 16S rRNA gene sequencing libraries

From the digestive-contents DNA, the prokaryotic DNA was targeted using specific primers amplifying the V3–V4 region (460 bp) of the 16S rRNA gene. The first step PCR was performed using 12.5 µL of KAPA HiFi HotStart Ready Mix (Roche, Boulogne-Billancourt, France), 5 µL of 1 µmol/L forward primer (5′-TCGTCGGCAGCGTCAGATGTGTATAAGAGACAGCCTACGGGNGGCWGCAG-3'), 5 µL of 1 µmol/L reverse primer (5'-GTCTCGTGGGCTCGGAGATGTGTATAAGAGACAGGACTACHVGGGTATCTAATCC-3'), and 2.5 µL of the extracted DNA (5 ng/µL). The thermocycling condition included a pre-incubation step of 3 min at 95 °C, 35 cycles of denaturation at 95 °C for 30 s, hybridization at 55 °C for 30 s, and elongation at 72 °C for 30 s, with a final extension step at 72 °C for 5 min. Controls, including *Escherichia coli* and ultra-pure water, were also added to the run. Additionally, DNA extracted from diet samples and water from the tanks were also included in the PCR. Amplification of the PCR products were confirmed using gel electrophoresis. The products were then sent to the genomic platform of Bordeaux (PGTB, Bordeaux, France). Libraries were prepared according to the standard protocol recommended by Illumina (Illumina, CA, USA). Index PCR was used to add the unique dual indexes to the sequences using the Nextera XT index kit according to the manufacturer’s recommendation (Illumina). The thermocycling conditions were the same as in step 1, except for PCR that was performed on 8 cycles. After PCR cleaning, libraries were quantified using the KAPA library quantification kit for Illumina platforms (Roche). Libraries were pooled at an equimolar concentration (4 nmol/L) and sequenced on a MiSeq platform using a 250 bp Paired End Sequencing Kit v2 (Illumina).

#### Sequence data analysis

The FROGS pipeline was used to perform the initial analysis of the sequence data [34]. The forward and reverse reads of each sample were merged, the adapter sequences were trimmed, and the sequences with long ambiguous bases (N) were removed (18.74% sequences withdrawn). After the quality filter steps, the resulting in 12,770,918 sequences, representing 81.26% of the initial input sequences were used for the downstream analysis. To group together amplicons with a maximum of one nucleotide difference between two amplicons, the clustering swarm protocol was used [35], resulting in the creation of 1,081,234 clusters. Clusters having an abundance less than 0.005% and not present in at least 4 samples were removed. This step resulted in 385 clusters. The PhiX database was used to remove the sequences corresponding to chloroplast and mitochondria [36]. The taxonomic affiliation was made with the silva138.1 pintail 100 16S reference database (https://www.arb-silva.de/documentation/release-138/). Finally, the samples had an average of 130,308 ± 24,495 sequences (minimum: 38,085; maximum: 194,676 sequences) with 239 OTUs (operational taxonomic units), ranging from 63 to 136 OTUs per sample. Microbiota data were all rarefied to 38,085 sequences/sample.

### Short-chain fatty acid measurement

The frozen intestinal content samples were weighed and placed into a 114-mL micro-chamber µCTE250 (Markes international, Llantrisant, UK). The micro-chamber was heated at 100 °C with a flow rate of 60 mL/min of dry nitrogen. A selected ion flow tube-mass spectrometer (SIFT-MS) was connected through a T connector to the micro-chamber with a sampling flow rate at 20 mL/min. A Voice 200 Ultra SIFT-MS (SYFT Technologies, Christchurch, New Zealand) generating three positive soft reagent ions (H_3_O^+^, O_2_^+^, and NO^+^) and with the nitrogen carrier gas (Air Liquid, Alphagaz 2) was used in this study. Full-scan mass spectra were recorded for each positive precursor ion in a range from 15 to 250 *m/z* with an integration time of 60 s and accumulated during 16 h. Identification was based on specific ion-molecule reaction patterns of target analytes with the three positive precursor ions described in the literature and in the database from LabSyft software (LabSyft 1.6.2, SYFT Technologies). Product ions from ion-molecule reactions of SCFAs are summarized in Additional file 2: Table S2. In SIFT-MS analysis, quantification is straight forward and requires only measurement of the count rate of the precursor ion [R] and product ions [P]. The analyte concentration in the flow tube [A] was determined according to the following formula:

$$\left[A\right]=\frac{\left[P\right]}{{t}_{r}k\left[R\right]}$$

Where *t*_*r*_ is the reaction time in the flow tube and *k* is the apparent reaction rate constant.

### Gene expression analysis

RNA from liver, and mid intestine, were extracted using TRIzol reagent (Invitrogen, Carlsbad, CA, USA). One µg of RNA was converted to cDNA with the superscript III reverse transcriptase enzyme (Invitrogen, Waltham, USA) and random primers (Promega, Madison, WI, USA). After the reverse transcription step, cDNA was diluted 80-fold for liver, and 40-fold for mid intestine, before being used in quantitative real-time PCR (qPCR). The RT-qPCR were performed in 384 well-plates in a C1000 Touchtm thermal cycler (BioRad, Hercules, CA, USA) using PerfeCTa SYBR green (VWR, Radnor, PA, USA). The total volume of reaction was 6 µL, including 2 µL of diluted cDNA mixed with 0.24 µL each of forward and reverse primer (10 µmol/L), 0.52 µL of RNase-free water, and 3 µL of SYBR green. Thermocycling conditions included a pre-incubation at 95 °C for 10 min, followed by 45 cycles of denaturation at 95 °C for 15 s, annealing at 60 °C for 10 s, and extension at 72 °C for 15 s. A Melt curve analysis was performed at the end of the last amplification cycle to confirm the specificity of the amplification reaction. Each RT-qPCR included replicate samples (duplicate of reverse transcription and PCR amplification), a standard curve in triplicate (a range of dilution of cDNA from a pool of all cDNA samples), and negative controls in duplicate (reverse transcriptase-free samples and RNA-free samples). The relative quantification of gene expression was carried out by the Bio-Rad CFX Maestro software (Version 4.0.2325.0418). Cq (Quantification cycle) values were further converted to relative quantities. Elongation factor 1 alpha (e*ef1a*) and beta-actin (*actb*) were used as reference for liver and mid intestine, respectively. The mRNA expression of the SCFA intestinal receptors (FFAR receptors) were analyzed using the new nomenclature described by Roy et al. [37]. The list of primers used in the present study are given in Additional file 3: Table S3.

### Hepatic enzymatic activities

The activity of the hepatic enzymes including Glucokinase, Pyruvate kinase, Glucose-6-phosphatase, and Fatty acid synthase were analyzed using the protocols described previously [6].

### Plasma immune markers

The lysozyme activity was measured in plasma according to the protocol described by Frohn et al. [38]. Total anti-protease activity was determined by assessing the ability of plasma to inhibit trypsin activity as described by Peixoto et al. [39]. Plasma nitric oxide content were assayed with the Nitrite/Nitrate, colorimetric test, following the manufacturer instructions (Roche, Boulogne-Billancourt, France).

### Statistical analysis

Zootechnical parameters, including initial and final body weight, specific growth rate, daily feed intake, feed efficiency, and protein efficiency ratio were calculated per tank (*n* = 3). The formula used are given in the legend of Table 2. The hepatosomatic index (liver weight × 100/fish weight) were obtained during the final sampling of the trial (*n* = 12 fish per condition/diet). All data are presented as mean ± standard deviation (SD). All statistical analyses were performed using the R software (version 4.0.3) [40]. Data were tested for normal distribution using the Shapiro–Wilk test and homogeneity of variance was tested using Bartlett’s test. Data were analyzed using a two-way ANOVA, with CHO/protein ratio and inulin as factors. Tukey’s HSD was used as post hoc test. When the two-way ANOVA not meet the assumptions of normality and homogenous variance, we used non parametric ANOVA. Results with a *P*-value < 0.05 were considered significant. The interactions between the 2 factors are identified by “CHO/protein × inulin”.

**Table 2:** Growth performances and feed utilization, in rainbow trout fed the experimental diets

**Zootechnical parameters**	**LS-0**	**LS-In**	**HS-0**	**HS-In**	***P*-values**		
					**CHO/protein**	**Inulin**	**Interaction**
Initial body weight, g	31.7 ± 0.26	31.9 ± 0.13	31.7 ± 0.19	31.8 ± 0.14			
Final body weight, g	168.21 ± 4.94	159.33 ± 5.28	170.58 ± 10.10	158.40 ± 3.69	NS	*	NS
Specific growth rate^a^, %/d	1.98 ± 0.04	1.91 ± 0.04	2.00 ± 0.07	1.91 ± 0.03	NS	*	NS
Daily feed intake^b^, g/fish/d	1.57 ± 0.03	1.49 ± 0.04	1.57 ± 0.07	1.53 ± 0.03	NS	NS (*P* = 0.058)	NS
Feed efficiency^c^	1.04 ± 0.02	1.02 ± 0.03	1.05 ± 0.04	0.99 ± 0.01	NS	*	NS
Protein efficiency ratio^d^	2.36 ± 0.04	2.32 ± 0.07	2.95 ± 0.12	2.78 ± 0.02	***	*	NS

The four feeding groups (LS-0: Low-starch with 0 inulin; LS-In: Low-starch with 2% inulin; HS-0: high-starch with 0 inulin; and HS-In: high-starch with 2% inulin) were given to 63 fish per group (3 tanks per group) for 12 weeks. Data are presented as mean ± SD (*n* = 3 tanks). Statistical differences were analyzed with a two-way ANOVA test and were considered statistically significant for *P* < 0.05

*NS* Not significant, *P < 0.05, ***P < 0.001

^a^Specific growth rate, %/d = 100 × [Ln (final body weight, g)–Ln (initial body weight, g)]/Experiment duration, d

^b^Daily feed intake, g/fish/d = Total feed consumed, g/Number of fish/Experiment duration, d

^c^Feed efficiency = (Final biomass, g − Initial biomass, g)/Total feed consumed, g

^d^Protein efficiency ratio = (Final biomass − Initial biomass)/Total protein consumed, g

The microbial composition was analyzed using the phyloseq package [41]. For alpha and beta diversity, data samples were rarified. Beta-diversity was analyzed with the Bray–Curtis distance using permutational multivariate analysis of variance (PERMANOVA) [42]. The mixOmics package was used to perform a Partial Least Square Discriminant Analysis (PLS-DA) to determine the most discriminant OTUs [43]. The rCCA (regularized canonical correlation analysis) function of the same package was used to understand the correlations between the bacterial OTUs and different host parameters.

## Results

### Zootechnical parameters, whole-body composition, and hepatic and plasma metabolites

A significant lower growth of the fish, resulting in a lower final body weight (*P* = 0.022) of the group fed with 2% inulin was observed after 12 weeks of feeding. This was accompanied by a lower specific growth rate (*P* = 0.017), and lower feed efficiency (*P* = 0.037). The protein efficiency ratio was positively affected (*P* = 1.459e-06) in rainbow trout fed with high-starch diets and negatively affected (*P* = 0.034) by inulin. Concerning the whole-body composition (Additional file 4: Table S4), no significant differences in the final values were observed for the dry matter (DM, %), ash (% of DM), protein level (% of DM), lipids level (% of DM), and gross energy (kJ/g of DM). The change in the CHO/protein ratio and the use of 2% inulin did not significantly affect plasma glucose levels (*P* > 0.05), presented in Table 3. In contrast, the triglycerides levels were significantly lower (*P* = 0.005) in the high starch groups independently of inulin (Table 3). A higher concentration of plasma lactate (*P* = 0.020) and a lower level of the cholesterol was measured (*P* = 0.003) in the high-starch groups (HS-0 and HS-In), than in the low-starch groups (LS-0 and LS-In). Regarding hepatic parameters, the factor starch induced a significant higher hepatosomatic index (*P* = 3.045e-08) with a concomitant increase of glycogen concentration (*P* = 7.916e-09).

**Table 3:** Plasma metabolites, hepatic glycogen contents, and hepato-somatic index in rainbow trout

	**LS-0**	**LS-In**	**HS-0**	**HS-In**	***P* values**		
					**CHO/protein**	**Inulin**	**Interaction**
Plasma parameters							
Glucose, g/L	1.13 ± 0.19	1.13 ± 0.17	1.26 ± 0.31	1.18 ± 0.20	NS	NS	NS
Triglycerides, g/L	4.00 ± 0.79	3.91 ± 0.79	3.42 ± 1.00	2.83 ± 0.97	^**^	NS	NS
Lactate, g/L	4.04 ± 1.48	3.52 ± 1.61	4.63 ± 1.52	5.14 ± 1.74	^*^	NS	NS
Cholesterol, g/L	2.63 ± 0.48	2.34 ± 0.48	1.94 ± 0.68	2.01 ± 0.60	^**^	NS	NS
Hepatic parameters							
Hepatosomatic index	0.91 ± 0.12	0.90 ± 0.09	1.17 ± 0.10	1.22 ± 0.13	^***^	NS	NS
Glycogen, mg/g	23.53 ± 16.32	20.72 ± 13.01	79.72 ± 33.00	71.91 ± 34.84	^***^	NS	NS

The four feeding groups (LS-0: Low-starch with 0 inulin; LS-In: Low-starch with 2% inulin; HS-0: high-starch with 0 inulin; and HS-In: high-starch with 2% inulin) were given to 63 fish per group (3 tanks per group) for 12 weeks. Data are presented as mean ± SD (*n* = 12 fish). Statistical differences were analyzed with a two-way ANOVA test and were considered statistically significant for *P* < 0.05

*NS* Not significant, *P < 0.05, **P < 0.01, ***P < 0.001

### Microbiota composition in mid intestines

**Fig. 2:**
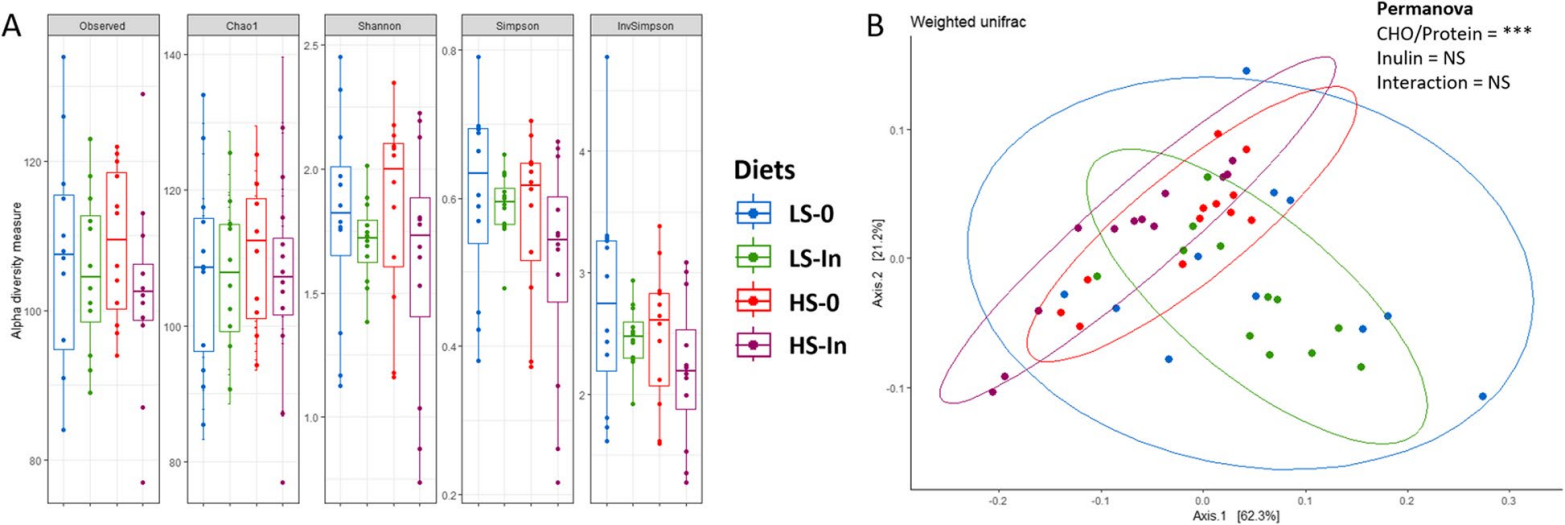
Bacterial alpha diversity (**A**) is represented in terms of observed OTUs, Chao1, Shannon, Simpson, InvSimpson, in the mid-intestinal section of rainbow trout after 12 weeks of feeding. Feeding groups are symbolized as LS-0: Low-starch with 0 inulin; LS-In: low-starch with 2% inulin; HS-0: High-starch with 0 inulin; and HS-In: High-starch with 2% inulin. Beta diversity (**B**) is presented by a PCoA representation (Bray–Curtis distance, Weighted-Unifrac analysis) in mid intestine section, according to the experimental diets. Beta diversity was compared using a pairwise PERMANOVA test and data were considered statistically different for *P* < 0.05. *n* = 12 fish per group

Modification of the CHO/protein ratio or the use of inulin did not affect significantly the alpha diversity (Fig. 2a), but the beta diversity was significantly (*P* = 0.003) affected by the change in the CHO/protein ratio (Fig. 2b).

**Fig. 3:**
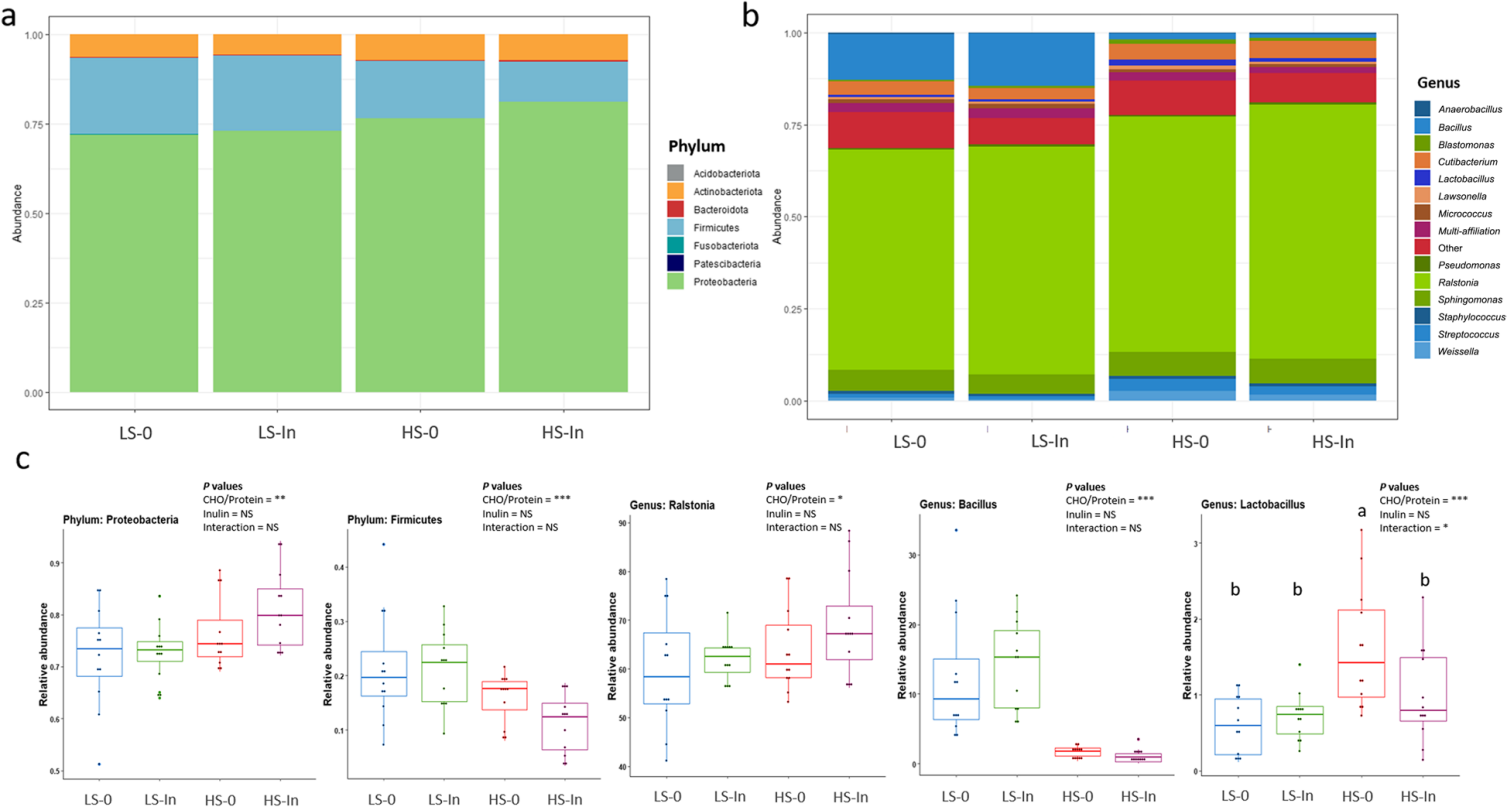
Microbial composition in the mid-intestinal section at phylum (**a**) and genus (**b**) levels after 12 weeks of feeding. In panel **b**, only the top 15 abundant genera are presented. Feeding groups are symbolized as LS-0: Low-starch with 0 inulin; LS-In: low-starch with 2% inulin; HS-0: High-starch with 0 inulin; and HS-In: High-starch with 2% inulin. On panel **c**, the relative abundance of the Proteobacteria and Firmicutes phyla and the relative abundances of the *Ralstonia*, *Bacillus*, and *Lactobacillus* genus are indicated. Statistical differences were analyzed with a two-way ANOVA test and were considered statistically significant for *P* < 0.05. Significant differences are represented by asterisk. ^*^P < 0.05, ^**^P < 0.01, ^***^P < 0.001. *n* = 12 fish per group

We identified 239 OTUs belonging to 7 phyla (Fig. 3a), and 100 genera (Fig. 3b). Proteobacteria (75.64% ± 4.09%), Firmicutes (17.48% ± 4.80%) and Actinobacteria (6.56% ± 0.82%) were the most abundant phyla among the groups. A significantly lower Firmicutes/Proteobacteria ratio (*P* = 0.007) (Additional file 5: Table S5) was observed for the high-starch groups due to a significant increase of relative abundance of Proteobacteria (+ 6.27% ± 2.65%, *P* = 0.006) and a significant decrease of the Firmicutes (−7.63% ± 3.14%, *P* = 0.001) in the high-starch groups (HS-0 and HS-In) (Fig. 3c). At the genus level, the most abundant genera in the Proteobacteria were the *Ralstonia* (63.67% ± 3.91%) and *Sphingomonas* (6.18% ± 0.70%) while in Firmicutes the most abundant genera were represented by *Bacillus* (7.33% ± 6.94%), *Streptococcus* (1.83% ± 0.78%), *Weissella* (1.37% ± 0.79%), *Enterococcus* (1.02% ± 0.55%), and *Lactobacillus* (0.99% ± 0.53%). Twenty-three genera were significantly affected either by the CHO/protein ratio or inulin (Table 4). The relative abundance of 15 bacterial genera were significantly increased by the high CHO/protein ratio: *Ralstonia*, *Sphingomonas*, *Cutibacterium*, *Streptococcus*, *Weissella*, *Blastomonas*, *Limosilactobacillus*, *Ligilactobacillus*, *Kocuria*, *Leuconostoc*, *Lacticaseibacillus*, *Brochothrix*, *Abiotrophia*, *Atopobium,* and *Kytococcus*. Among them, 9 genera belonging to the lactic-acid bacteria (LAB) were increased by the high-starch diet. While 5 genera were decreased by the factor starch: *Bacillus*, *Enterococcus*, *Lactococcus*, *Floricoccus*, *Aneurinibacillus*, including 2 LAB. Finally, only four genera were affected by inulin, three of them showing a lower proportion: *Streptococcus*, *Weissella*, and *Peptoniphilus* while higher proportion of *Porphyrobacter* were observed. Finally, CHO/protein × inulin interaction was found for *Lactobacillus* genus: the use of 2% inulin led to an increase of the proportion in the low-starch groups (LS-0, LS-In), and a decrease of the proportion in high-starch groups (Fig. 3c).

**Table 4:** Abundances of different genera significantly affected by experimental diets

**Genera, %**	**LS-0**	**LS-In**	**HS-0**	**HS-In**	***P* values**		
					**CHO/protein**	**Inulin**	**Interaction**
*Ralstonia*	59.74 ± 12.13	62.11 ± 4.40	63.88 ± 8.55	68.96 ± 10.82	^*^	NS	NS
*Bacillus*	12.45 ± 9.21	14.16 ± 6.40	1.66 ± 0.82	1.05 ± 1.02	^***^	NS	NS
*Sphingomonas*	5.78 ± 2.16	5.43 ± 1.61	6.53 ± 1.41	6.97 ± 2.22	z	NS	NS
*Cutibacterium*	3.77 ± 1.45	3.09 ± 1.18	4.29 ± 1.71	4.65 ± 1.96	^*^	NS	NS
*Streptococcus*	1.09 ± 0.46	0.76 ± 0.31	3.15 ± 1.06	2.33 ± 1.29	^***^	^*^	NS
*Weissella*	0.76 ± 0.68	0.44 ± 0.20	2.65 ± 1.14	1.64 ± 1.12	^***^	^*^	NS
*Enterococcus*	1.52 ± 0.96	1.69 ± 0.68	0.49 ± 0.19	0.39 ± 0.35	^***^	NS	NS
*Lactobacillus*	0.62 ± 0.38^b^	0.72 ± 0.31^b^	1.62 ± 0.81^a^	0.99 ± 0.62^b^	^***^	NS	^*^
*Blastomonas*	0.59 ± 0.18	0.51 ± 0.29	1.07 ± 0.56	0.79 ± 0.28	^***^	NS	NS
*Limosilactobacillus*	0.43 ± 0.25	0.37 ± 0.25	0.72 ± 0.41	0.7 ± 0.49	^**^	NS	NS
*Ligilactobacillus*	0.26 ± 0.31	0.22 ± 0.27	0.52 ± 0.55	0.51 ± 0.58	^*^	NS	NS
*Kocuria*	0.17 ± 0.17	0.17 ± 0.15	0.27 ± 0.2	0.31 ± 0.23	^*^	NS	NS
*Lactococcus*	0.19 ± 0.14	0.22 ± 0.17	0.11 ± 0.20	0.03 ± 0.06	^**^	NS	NS
*Leuconostoc*	0.08 ± 0.13	0.03 ± 0.05	0.21 ± 0.24	0.12 ± 0.14	^*^	NS	NS
*Floricoccus*	0.11 ± 0.11	0.20 ± 0.10	0.06 ± 0.10	0.03 ± 0.11	^***^	NS	NS
*Porphyrobacter*	0.04 ± 0.05	0.09 ± 0.07	0.08 ± 0.11	0.13 ± 0.09	NS	*	NS
*Peptoniphilus*	0.17 ± 0.23	0.05 ± 0.07	0.09 ± 0.11	0.02 ± 0.04	NS	^*^	NS
*Lacticaseibacillus*	0 ± 0	0.01 ± 0.02	0.03 ± 0.05	0.03 ± 0.05	^*^	NS	NS
*Brochothrix*	0 ± 0	0.01 ± 0.02	0.02 ± 0.03	0.03 ± 0.05	^*^	NS	NS
*Aneurinibacillus*	0.02 ± 0.03	0.03 ± 0.06	0 ± 0	0 ± 0	^**^	NS	NS
*Abiotrophia*	0.01 ± 0.01	0 ± 0	0.03 ± 0.04	0.02 ± 0.03	^*^	NS	NS
*Atopobium*	0.01 ± 0.01	0 ± 0	0.01 ± 0.01	0.03 ± 0.05	^*^	NS	NS
*Kytococcus*	0 ± 0	0 ± 0	0.01 ± 0.02	0.02 ± 0.04	^*^	NS	NS

The four feeding groups (LS-0: Low-starch with 0 inulin; LS-In: Low-starch with 2% inulin; HS-0: high-starch with 0 inulin; and HS-In: high-starch with 2% inulin) were given to 63 fish per group (3 tanks per group) for 12 weeks. Data are presented as mean ± SD (*n* = 12 fish). Statistical differences were analyzed with a two-way ANOVA test and were considered statistically significant for *P* < 0.05

In case of interaction, a post-hoc Tukey’s test was performed (*P* < 0.05). ^a,b^Different superscripts indicated significant differences between groups

*NS* Not significant, *P < 0.05, **P < 0.01, ***P < 0.001

**Fig. 4:**
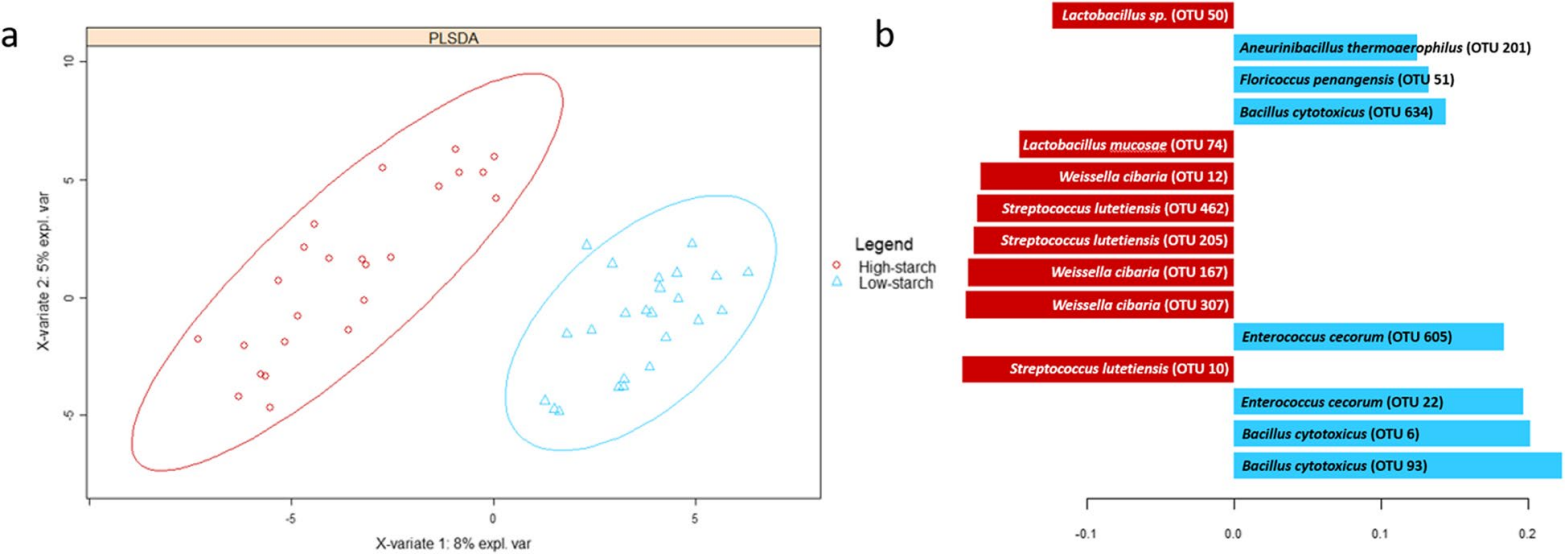
PLS-DA analysis for fish fed the high-starch and low-starch diets (independently of inulin) based on OTUs abundance (**a**). Each red points or blue triangles represent a sample. Samples can be discriminated according to experimental group on component 1. Contribution level of the top 15 OTUs are presented (**b**). Red bars correspond to the high-starch group and blue bars to the low-starch group. *n* = 12 fish per group

The change in the CHO to protein ratio induced a clear separation of the bacterial communities, in PLS-DA (Fig. 4a). The most discriminant OTUs within high-starch group (Fig. 4b), were *Streptococcus lutetiensis* (OTU 10, 205, 462), *Weissella cibaria* (OTU 307, 167, 12), *Lactobacillus mucosae* (OTU 74), and *Lactobacillus* sp. (OTU 50). In contrast, in the low-starch group, *Bacillus cytotoxicus* (OTU 93, 6, 634), *Enterococcus cecorum* (OTU 22, 605), *Floricoccus penangensis* (OTU 51), and *Aneurinibacillus thermoaerophilus* (OTU 201) were the most discriminant.

### Short-chain fatty acids in mid intestines digestive contents

**Fig. 5:**
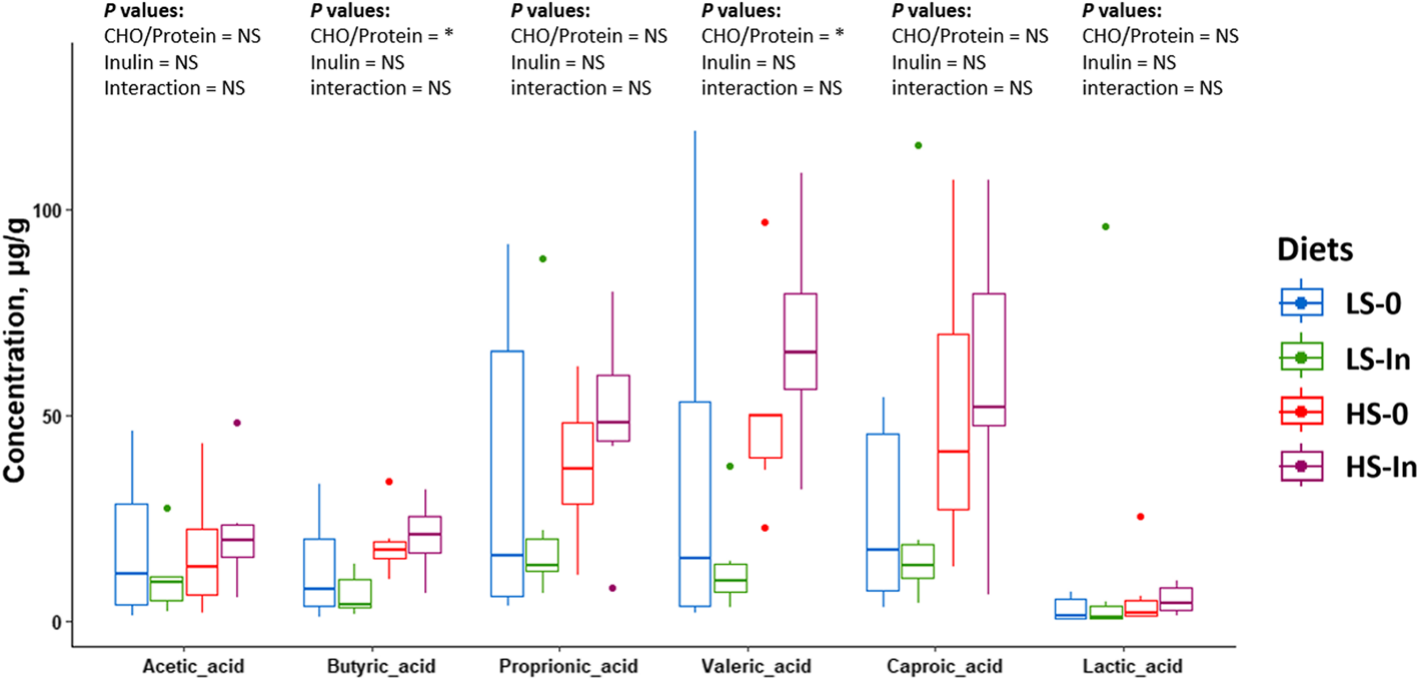
Levels of acetic acid, butyric acid, propionic acid, valeric acid, caproic acid as well as lactic acid were measured (µg) relative to the mass of the mid-intestinal digestive contents (g) with SIFT-MS mass spectrometry. Feeding groups are symbolized as LS-0: Low-starch with 0 inulin; LS-In: low-starch with 2% inulin; HS-0: High-starch with 0 inulin; and HS-In: High-starch with 2% inulin. Statistical differences were analyzed with a two-way ANOVA test and were considered statistically significant for *P* < 0.05. Significant differences are represented by an asterisk. ^*^P < 0.05, ^**^P < 0.01, ^***^P < 0,001. *n* = 6 fish per group

A significant increase of butyric acid (*P* = 0.014), and valeric acid (*P* = 0.011) were detected in the high-starch groups while no significant differences were detected for acetic, propionic, and caproic acids, as well as the lactic acid (Fig. 5).

### Expression of genes involved in liver metabolism

#### Liver

A two-way ANOVA revealed that there was a significant interaction (CHO/protein × inulin) effect on the expression of *glut2b*, a gene involved in glucose transport (Table 5). Glucokinase paralogue *gcka* expression was significantly higher (*P* = 7.42e-08) in high starch groups while a significant interaction effect was observed for the expression of the second paralogue *gckb* (*P* = 1.06e-09). We also detected an interaction effect on the *pfkIa*, *pfkIb* gene expression and *pk* expression. These interactions showed an up-regulation of these genes with 2% inulin in the low-starch groups and a down-regulation in high-starch groups. Regarding the gluconeogenesis pathway, the expression of *pck1* (*P* = 0.04) and *fbp1b1* (*P* = 0.0024) genes were significantly down-regulated in the two high-starch groups (HS-0 and HS-In). A significant interaction was detected for both *fbp1b1* and *g6pcb2a* genes. This interaction showed a higher expression in the low-starch groups with 2% inulin and a lower expression in the high-starch groups with 2% inulin. Finally, only the *fbp1a* gene was significantly affected by inulin. Regarding lipogenesis pathway, significant interactions were detected for mRNA levels of *serbf1* (*P* = 4.50e-04), *aclyb* (*P* = 1.2e-03), and *aclyc* (*P* = 3.2e-04). These genes were indeed up-regulated with 2% inulin in the low-starch and down-regulated in the high-starch groups. High CHO/protein levels induced a significant decrease of the *aca-ba* gene expression. The high CHO/protein level led to a down-regulation of the *srepb2b*, *hmgcrb*, *dhcr7a* genes.

**Table 5:** The mRNA levels of genes involved in different metabolic pathways in liver

**Liver gene expression**		**LS-0**	**LS-In**	**HS-0**	**HS-In**	***P*** **values**		
						**CHO/protein**	**Inulin**	**Interaction**
Glucose transport	*glut2a*	0.75 ± 0.46	1.11 ± 0.50	1.16 ± 1.13	0.70 ± 0.63	NS	NS	NS
	*glut2b*	0.79 ± 0.71^b^	2.12 ± 1.67^a^	1.13 ± 0.65^ab^	0.82 ± 0.86^b^	NS	NS	^**^
Glycolysis	*gcka*	0.24 ± 0.35	2.59 ± 4.09	17.89 ± 8.13	12.07 ± 10.43	^***^	NS	NS
	*gckb*	0.27 ± 0.58^c^	1.53 ± 1.34^bc^	15.03 ± 7.15^a^	5.58 ± 3.86^b^	^***^	^**^	^***^
	*pfkla*	1.03 ± 1.05^b^	2.71 ± 2.30^a^	1.13 ± 0.62^b^	0.73 ± 0.72^b^	^*^	NS	^*^
	*pfklb*	0.97 ± 0.81^b^	2.43 ± 2.15^a^	1.33 ± 0.75^ab^	0.84 ± 0.85^b^	NS	NS	^*^
	*pk*	0.93 ± 0.81^b^	2.23 ± 1.56^a^	1.05 ± 0.74^b^	0.67 ± 0.55^b^	^*^	NS	^**^
Gluconeogenesis	*pck1*	1.85 ± 1.62	2.26 ± 2.42	0.98 ± 1.26	0.94 ± 1.38	^*^	NS	NS
	*pck2*	1.26 ± 1.31	2.09 ± 2.22	1.44 ± 0.80	0.96 ± 1.32	NS	NS	NS
	*fbp1a*	0.59 ± 0.52	1.72 ± 1.44	0.76 ± 0.81	1.13 ± 1.48	NS	^*^	NS
	*fbp1b1*	1.11 ± 1.21^ab^	2.32 ± 1.68^a^	0.76 ± 0.45^b^	0.59 ± 0.68^b^	^**^	NS	^*^
	*fbp1b2*	1.35 ± 1.71	1.97 ± 1.47	1.01 ± 0.76	0.46 ± 0.38	^*^	NS	NS
	*g6pca*	1.07 ± 1.18	2.45 ± 2.01	1.20 ± 0.83	1.01 ± 1.10	NS	NS	NS
	*g6pcb1b*	1.09 ± 0.70	1.31 ± 1.28	1.28 ± 1.00	1.32 ± 1.25	NS	NS	NS
	*g6pcb2a*	0.59 ± 0.65^b^	2.75 ± 2.58^a^	2.55 ± 2.08^ab^	0.88 ± 1.23^ab^	NS	NS	^**^
Lipogenesis	*srebf1*	0.61 ± 0.77^b^	2.77 ± 2.10^a^	1.90 ± 1.34^ab^	0.89 ± 1.27^b^	NS	NS	^***^
	*g6pdb*	0.67 ± 0.66	2.11 ± 1.75	1.07 ± 0.75	1.02 ± 1.49	NS	NS	NS
	*aclyb*	0.76 ± 0.66^b^	1.60 ± 1.03^ab^	2.16 ± 1.37^a^	0.80 ± 1.01^b^	NS	NS	^**^
	*aclyc*	0.85 ± 0.81^b^	2.76 ± 2.33^a^	2.18 ± 1.29^ab^	0.75 ± 0.52^ab^	NS	NS	^***^
	*aca-aa*	1.20 ± 1.72	2.67 ± 2.74	3.28 ± 2.89	2.65 ± 3.29	NS	NS	NS
	*aca-ba*	1.59 ± 1.37	2.84 ± 2.27	0.84 ± 0.53	0.80 ± 0.75	^**^	NS	NS
	*fasna*	0.81 ± 0.48	1.06 ± 0.56	1.43 ± 1.17	1.37 ± 1.70	NS	NS	NS
	*fasnb*	1.24 ± 1.00	1.40 ± 0.503	1.25 ± 0.81	1.22 ± 1.06	NS	NS	NS
Cholesterol biosynthesis	*srepb2a*	1.05 ± 1.15	1.79 ± 1.20	1.19 ± 0.59	1.06 ± 1.15	NS	NS	NS
	*srepb2b*	1.04 ± 0.90	1.70 ± 1.18	0.88 ± 0.35	0.70 ± 0.63	^*^	NS	NS
	*hmgcra*	0.94 ± 0.74	1.19 ± 0.80	1.11 ± 0.85	1.01 ± 1.12	NS	NS	NS
	*hmgcrb*	1.58 ± 1.21	1.26 ± 0.84	0.92 ± 0.59	0.70 ± 0.63	^*^	NS	NS
	*dhcr7a*	1.34 ± 0.99	1.43 ± 1.09	0.77 ± 0.59	0.76 ± 0.78	^*^	NS	NS
	*dhcr7b*	0.99 ± 0.41	1.16 ± 0.85	0.49 ± 0.22	0.85 ± 0.78	NS	NS	NS

The four feeding groups (LS-0: Low-starch with 0 inulin; LS-In: Low-starch with 2% inulin; HS-0: high-starch with 0 inulin; and HS-In: high-starch with 2% inulin) were given to 63 fish per group (3 tanks per group) for 12 weeks. Data are presented as mean ± SD (*n* = 12 fish). Statistical differences were analyzed with a two-way ANOVA test and were considered statistically significant for *P* < 0.05

In case of interaction, a post-hoc Tukey’s test was performed (*P* < 0.05). ^a,b^Different superscripts indicated significant differences between groups. *glut2a* and *glut2b*: glucose transporter paralogs. *gcka* and *gckb*: glucokinase paralogs. *pfkla* and *pfklb*: 6-phosphofructokinase paralogues. *Pk*: pyruvate kinase. *pck1* and *pck2*: phosphoenolpyruvate carboxykinase paralogs. *fbp1a*, *fbp1b1,* and *fbp1b2*: fructose 1.6-bisphosphatase paralogues. *g6pca*, *g6pcb1b,* and *g6pcb2a*: glucose-6-phosphatase paralogs. *srebf1:* sterol regulatory element binding factor 1. *g6pdb*: glucose-6-phosphatase dehydrogenase. *aclyb,* and *aclyc*: adenosine triphosphate citrate lyase paralogues. *aca-aa* and *aca-ba*: acetylcoA carboxylase paralogues. *fasna* and *fasnb*: fatty acid synthase paralogs. *srebp-2a* and *srebp-2b*: sterol regulatory element-binding protein 2 paralogues. *hmgcra*, and *hmgcrb*: hydroxymethylglutaryl-CoA synthase paralogues. *dhcr7a,* and *dhcr7b*: 7-dehydrocholesterol reductase paralogues

*NS* Not significant, *P < 0.05, **P < 0.01, ***P < 0.001

#### Mid-intestine

The mRNA expression of the SCFA intestinal receptors (FFAR receptors) were analyzed in mid-intestine (Table 6). We observed that the high CHO/protein levels resulted in a higher expression of every gene coding for *ffar*. Conversely, inulin did not affect the expression of these genes.

**Table 6:** mRNA levels of free fatty acid receptors (FFAR) in mid-intestine

**Free fatty acid receptors**	**LS-0**	**LS-In**	**HS-0**	**HS-In**	***P* values**		
					**CHO/protein**	**Inulin**	**Interaction**
*ffar1*	1.51 ± 1.16	1.05 ± 1.20	0.48 ± 0.46	0.50 ± 0.40	^**^	NS	NS
*ffar2a1a*	1.52 ± 1.06	1.15 ± 1.28	0.57 ± 0.59	0.56 ± 0.39	^**^	NS	NS
*ffar2a2*	1.63 ± 1.23	1.14 ± 1.39	0.34 ± 0.26	0.47 ± 0.32	^***^	NS	NS
*ffar2b1.1*	1.36 ± 1.01	1.13 ± 1.37	0.41 ± 0.43	0.51 ± 0.40	^**^	NS	NS
*ffar2b1.2*	1.61 ± 1.18	1.12 ± 1.45	0.52 ± 0.52	0.63 ± 0.38	^**^	NS	NS
*ffar2b2a*	1.65 ± 1.21	1.14 ± 1.57	0.42 ± 0.42	0.48 ± 0.29	^**^	NS	NS
*ffar2b2b1*	1.82 ± 1.40	1.03 ± 1.32	0.41 ± 0.28	0.53 ± 0.37	^**^	NS	NS

The four feeding groups (LS-0: Low-starch with 0 inulin; LS-In: Low-starch with 2% inulin; HS-0: high-starch with 0 inulin; and HS-In: high-starch with 2% inulin) were given to 63 fish per group (3 tanks per group) for 12 weeks. Data are presented as mean ± SD (*n* = 12 fish). Statistical differences were analyzed with a two-way ANOVA test and were considered statistically significant for *P* < 0.05

*NS* Not significant, **P < 0.01, ***P < 0.001

### Hepatic enzymatic activities

**Fig. 6:**
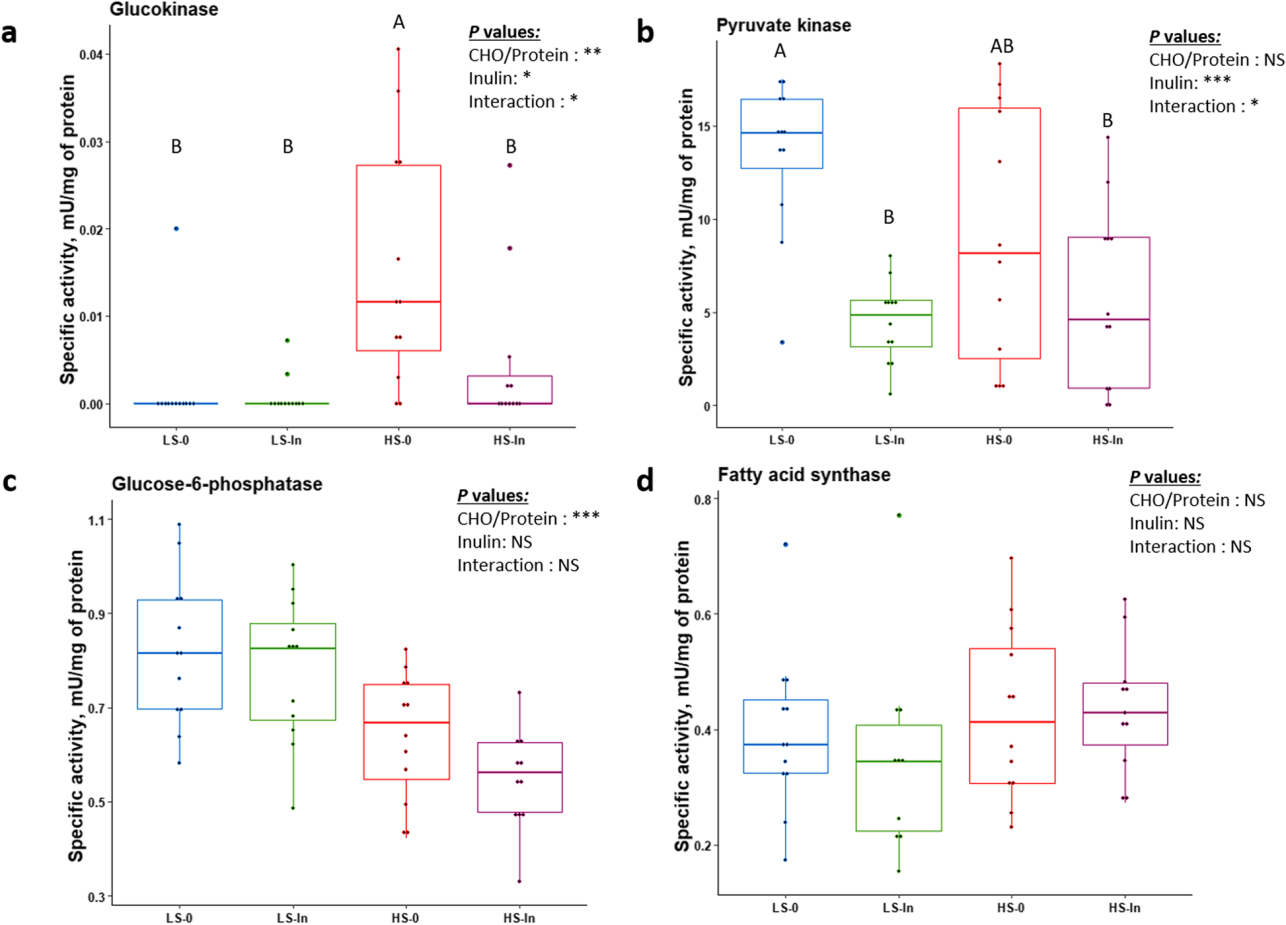
Hepatic enzymatic activities of glucokinase (**a**), pyruvate kinase (**b**), glucose-6-phosphatase (**c**), and fatty-acid synthase (**d**) after 12 weeks of feeding. Enzymatic activities were measured in liver samples relative to the average of milligram of proteins. Feeding groups are symbolized as LS-0: Low-starch with 0 inulin; LS-In: low-starch with 2% inulin; HS-0: High-starch with 0 inulin; and HS-In: High-starch with 2% inulin. Statistical differences were analyzed with a two-way ANOVA test and were considered statistically significant for *P* < 0.05. Significant differences are represented by an asterisk. ^*^P < 0.05, ^**^P < 0.01, ^***^P < 0.001. *n* = 12 fish per group

We detected a CHO/protein: inulin interaction for both glucokinase and pyruvate kinase (Fig. 6a and b). The glucose-6-phosphatase activity was decreased significantly due to high starch (*P* = 8.806e-05) (Fig. 6c). The key enzyme involved in lipogenesis, fatty acid synthase (FAS), was not affected by any of the factors or the interaction between them (Fig. 6d).

### Immune response to the starch and inulin factors

#### Liver

**Fig. 7:**
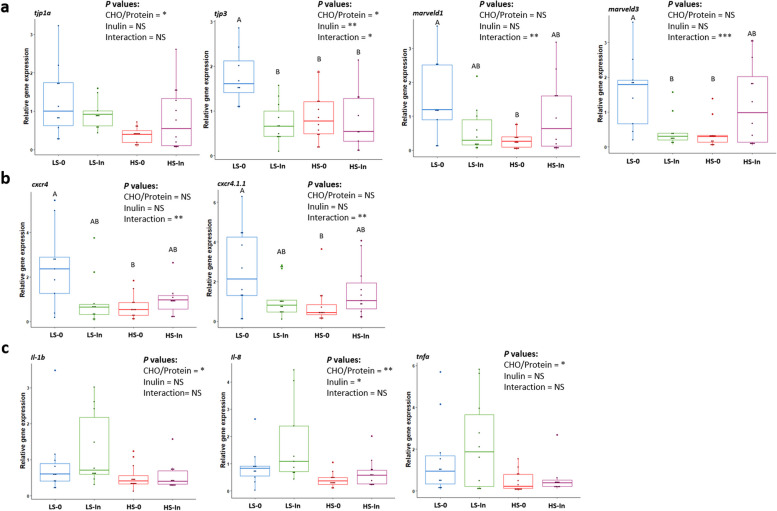
Mid-intestinal mRNA expression levels of tight-junction protein associated genes (*tjp1a*, *tjp3*, *marveld1*, and *marveld3*) (**a**), mid-intestine mRNA expression levels of C-X-C motif chemokine receptor 4 paralogues (*cxcr4* and *cxcr4.1.1*) (**b**), *il1b*, *il8*, and *tnfa* (**c**) after 12 weeks of feeding. Feeding groups are symbolized as LS-0: Low-starc h with 0 inulin; LS-In: low-starch with 2% inulin; HS-0: High-starch with 0 inulin; and HS-In: High-starch with 2% inulin. Statistical differences were analyzed with a two-way ANOVA test and were considered statistically significant for *P* < 0.05. Significant differences are represented by an asterisk. ^*^P < 0.05, ^**^P < 0.01, ^***^P < 0.001. *n* = 12 fish per group

High starch diets (HS-0, HS-In) had significantly lowered the expression of *il1b*, *il8*, and *tnfa* (*il1b*: *P* = 0.014; *il8*: *P* = 0.004; *tnfa*: *P* = 0.016) (Fig. 7c). In addition, the expression of *il8* was also higher in the 2% inulin groups (LS-In, HS-In) (*P* = 0.040).

#### Intestine

A significant interaction between the factors starch and inulin was observed for the expression of two genes related to chemokine receptors (*cxcr4*: *P* = 8.18e-03, *cxcr4.1.1*: *P* = 9.82e-03) (Fig. 7b). Moreover, the expression of four genes coding for tight junction proteins, *tjp1a*, *tjp3*, *marveld1* and *marveld3*, were significantly affected by the diets. A significant decrease of the expression of *tjp1a* (*P* = 1.56e-03) was evident in the high-starch group and a CHO/protein × inulin interaction effect was observed on the expression of *tjp3 (P* = 1.3e-02), *marveld1* (*P* = 4.16e-03) and *marveld3* (*P* = 6.00e-04) genes (Fig. 7a).

#### Plasma

**Fig. 8:**
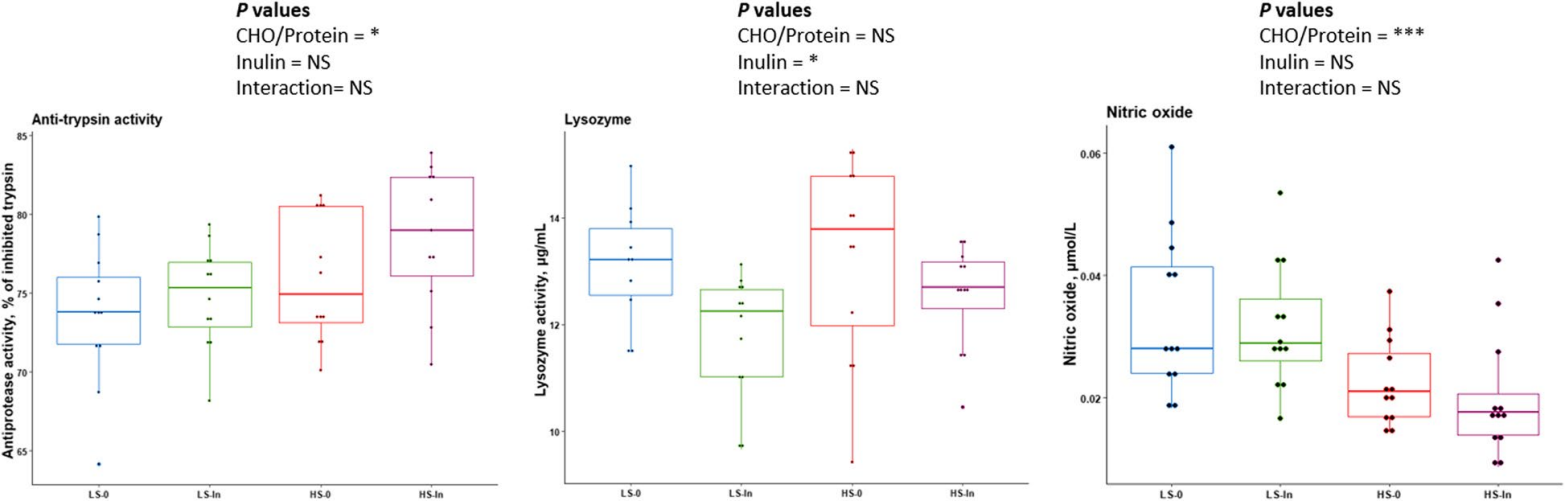
Plasma immune markers after 12 weeks of feeding. Feeding groups are symbolized as LS-0: Low-starch with 0 inulin; LS-In: low-starch with 2% inulin; HS-0: High-starch with 0 inulin; and HS-In: High-starch with 2% inulin. Data are presented as the mean ± SD. Statistical differences were analyzed with a two-way ANOVA test and were considered statistically significant for *P* < 0.05. Significant differences are represented by an asterisk. ^*^P < 0.05, ^**^P < 0.01, ^***^P < 0.001. *n* = 12 fish per group

In the high-starch groups (HS-0 and HS-In), we observed that antiprotease activity (Fig. 8) was significantly increased (*P* = 0.013) and the plasma nitric oxide concentration was significantly decreased (*P* = 0.001). Inulin dietary supplementation decreased the plasma lysozyme activity (*P* = 1.258e-02).

### Correlation between OTU abundances with immune and metabolic parameters

**Fig. 9:**
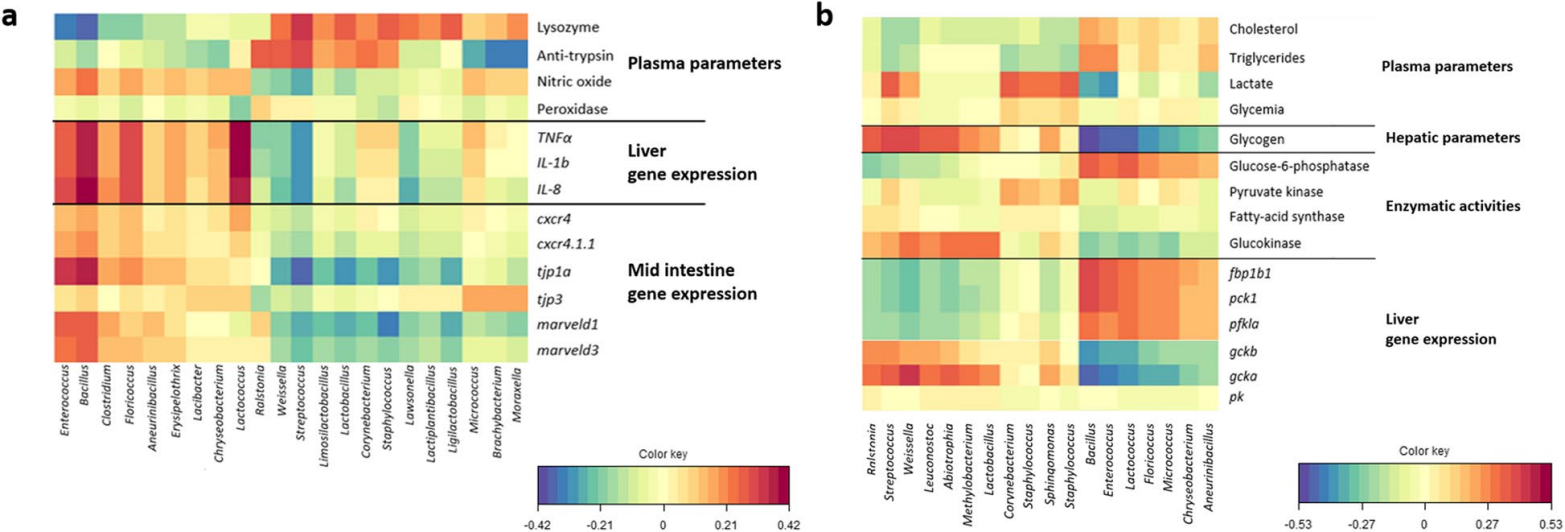
Correlations between OTUs abundances and immune markers (**a**), and metabolic parameters (**b**). Heatmap correlations, were calculated using regularized canonical correlation analysis (rCCA). *n* = 12 fish per group

We observed that the *Enterococcus*, *Bacillus*, *Floricoccus*, and *Lactococcus* were positively correlated with the expression of some hepatic cytokines (*tnfa*, *il1b*, *il8*) as well as the expression of tight junction protein (*tjp1a, tjp3, marveld1, marveld3*) coding genes in the mid intestine (Fig. 9a). Conversely, the genera *Weissella*, *Streptococcus*, *Limosilactobacillus*, *Lactobacillus*, *Corynebacterium*, *Staphylococcus*, *Lawsonella*, *Lactiplantibacillus*, *Ligilactobacillus*, and *Moraxella* were positively correlated with the lysozyme and antiprotease plasma activities. Finally, *Ralstonia* genera was positively correlated with the plasma antiprotease activity.

*Ralstonia*, *Streptococcus*, *Weissella*, *Leuconostoc*, *Abiotrophia*, *Methylobacterium* and *Lactobacillu*s were positively correlated the expression of *gcka* gene, the enzymatic activity of glucokinase and glycogen level in the liver (Fig. 9b). *Corynebacterium*, *staphylococcus*, *Sphingomonas*, and *Staphylococcus* were positively correlated with plasma lactate concentration. Conversely, *Bacillus*, *Enterococcus*, *Lactococcus*, *Floricoccus*, *Micrococcus*, *Chryseobacterium* and *Aneurinibacillus* were positively associated with the feed efficiency, plasma metabolites (cholesterol, lactate and triglycerides), as well as the enzymatic activity of glucose-6-phosphatase in the liver, and the expression of *fbp1b1*, *pck1*, and *pfkla* genes in liver.

## Discussion

The sustainability of aquaculture and the development of plant-based diets for aquafeeds has led to extensive research [44–46]. In this context, we have recently shown that the use of a high-starch diet in a 100% plant-based diet has no adverse effects on the rainbow trout metabolism, but does not improve growth [6]. Interestingly, the use of prebiotics such as inulin has already been investigated in teleosts and could have beneficial effects associated with a high-starch diet [31], but little is known about its effect on host metabolism and immunity, in contrast to mammals [47, 48]. In this study, we wanted to understand the effects of inulin supplementation in rainbow trout fed a diet containing either high or low CHO/protein ratio, with the basal diet comprising of 100% plant-based ingredients. Thus, we analyzed the intestinal microbiota, the host metabolism, growth parameters, and some immune markers.

The effect of inulin (or non-digestible polysaccharides) is mainly mediated by the intestinal microbiome via the production of bacterial metabolites such as SCFA or lactate [49, 50]. Moreover, a high CHO/protein ratio can affect the rainbow trout gut microbiota and in particular the Firmicutes/Proteobacteria ratio as well as the lactic-acid bacteria [6]. In our study, the intestinal microbiota was dominated by Proteobacteria and Firmicutes regardless the diets, as previously described in salmonids [2, 3, 6, 51–54]. Interestingly, in humans, the Firmicutes phyla is specialized in the degradation of non-digestible polysaccharides [8, 55]. At genus level, *Ralstonia* (from Proteobacteria) dominates the gut microbiota. *Ralstonia* has already been described in the intestinal communities of rainbow trout [3, 56], tilapia, goldfish (*Carassius auratus*) and largemouth bass (*Micropterus salmoides*) [57–59]. However, in salmonids, other genera, such as *Photobacterium* or *Vibrio* belonging to the Proteobacteria are found in high abundance in the intestinal microbiota [52, 53]. Interestingly, we observed that the intestinal microbiota has been strongly modified by the change in the CHO/protein ratio in the fish fed with a 100% plant-based diet as previously shown by Defaix et al. [6]. Indeed, the relative abundances of Proteobacteria and Firmicutes phyla were strongly affected by the change in the CHO/protein ratio, confirmed by the decrease of the Firmicutes/Proteobacteria ratio.

In the high-starch groups, the decrease in the Firmicutes/Proteobacteria ratio may seems surprising since the level of digestible carbohydrates is elevated in these groups and it is known that many bacteria belongings to the Firmicutes are known to have the ability to encode for carbohydrate-active enzymes allowing the degradation of polysaccharides [60]. However, this decrease may be associated with the significant lower abundances of the *Bacillus* genus in the high-starch groups. Although in fish, some species of bacilli such as *Bacillus cereus*, *Bacillus subtilis*, *Bacillus amyloliquefaciens* are known to degrade polysaccharides [61], bacilli found in this study such as *Bacillus cytotoxicus* in particular in low-starch groups (Fig. 4b) has not been demonstrated. Conversely, in the high-starch groups, many lactic acid bacteria belonging to the Firmicutes groups were observed in higher proportion. Interestingly, these bacteria are known to metabolize dietary plant glucosides and externalizes their bioactive phytochemicals [62]. Additionally, these LAB were found in higher proportion in rainbow trout fed with plant-based diet in comparison to a diet containing fishmeal and fish oil [63]. These LAB, in particular *Lactobacillus, Lactococcus* and *Streptococci*, can present a symbiotic relationship with the host [64] and are able to produce lactate through homolactic acid fermentation [64, 65]. Interestingly, two SCFAs, butyric acid and valeric acid, were found to be higher in high starch groups according to previous work [6]. The production of key cross-talk molecules such as SCFAs, in particular butyric acid, may have several beneficial effects on the fish gut health and are known to improve immunity [11, 66], and an enhancement of the glucose homeostasis [31]. In contrast, we observed that the microbiota of trout fed with the low CHO/protein ratio presented a lower level of LAB and higher proportion of *Enterococcus* and *Bacillus cytotoxicus,* which could be opportunistic pathogens [67, 68] and could have detrimental effects on trout gut health. There were also some effects of inulin on the intestinal microbiota. Indeed, four genera were modified by the use of inulin in contrast to previous studies on fish where inulin strongly affected the gut microbiota including rainbow trout [69], and Nile tilapia [31]. Concerning *Lactobacillus*, a CHO/protein × inulin interaction was observed. This result requires further investigations to elucidate the potential role of inulin. Additionally, we did not record a significant change in the SCFAs and lactic acid production with the use of inulin unlike a previous work in tilapia where the authors observed an increase of SCFA production in tilapia associated with a high-starch diet [31].

In order to detect if the higher production of SCFA in the group fed with high CHO/protein ratio can act on the host metabolism, we studied the expression of the FFAR encoding genes on the mid-intestine. Indeed, these G protein-coupled receptors participate in both immune and metabolic regulation after activation by SCFA [70]. In humans, the activation of FFAR lead to signal molecules production (Gα_q/11_ or Gα_i/o_) enhancing the secretion of insulin by β-pancreatic cells [71]. These classes of receptors have already been characterized in the rainbow trout intestine, and were found to be regulated when the fish were fed with a plant-based diet [2, 37]. Interestingly, we observed that all the FFAR receptors (*ffar1*, *ffar2a1*, *ffar2a1a*, *ffar2b1a*, *ffar2b1b*, *ffar2b2a*, and *ffar2b2b1)* in mid-intestine were significantly down-regulated by the high CHO/protein ratio. Inulin had no impact on the expression of these genes. In a previous study, the *ffar2b1a* (previously *ffar31*) expression was significantly increased in trout fed a 100% plant-based diet with inulin [2]. Moreover, it is known that the chronic stimulation (here during 12 weeks of feeding) of G protein-coupled receptors lead to the recruitment of β-arrestins preventing further stimulation of the downstream signaling pathways [72, 73]. In mid intestine, the decrease in the expression of these key receptors therefore probably reflects the desensitization (even remains to be demonstrated in fish) of these receptors after being stimulated by SCFA, demonstrating the high reactivity of FFARs in the presence of endogenous ligands in rainbow trout in mid intestine.

Increasing the CHO proportion by decreasing the proportion of plant protein in the high-starch diet did not affect the final weight of fish but resulted in an increase of the protein efficiency ratio as shown in our previous study [6]. CHO could prevent the protein catabolism for energy needs, as shown previously in fish fed with marine ingredients [74], and now in trout with plant based raw material [6]. But, unexpectedly, at the end of the 12-week feeding, rainbow trout with a diet supplemented with 2% inulin caused a lower specific growth rate, lower final body weight, and lower feed efficiency. Conversely, previous studies on rainbow trout did not observe a decrease of growth when fish were fed with 2% inulin in a 100% plant-based diet [2, 3].

Rainbow trout are known to be “low users” of CHO when fed with a FM/FO diet. High levels of CHO generally lead to a persistent post-prandial hyperglycemia [75–77], explained in part, by a deregulation of gluconeogenesis pathways [78]. In this study, the high-starch diet, in combination with a 100% plant-based diet, did not induce post-prandial hyperglycemia, suggesting an efficient glucose homeostasis, which we previously observed in trout fed a 100% plant-based diet [6]. Interestingly, the use of these 2-factors experiments led to many significant interactions at molecular levels such as the glycolysis, gluconeogenesis, and lipogenesis pathways. Indeed, we observed an up-regulation of multiple genes in fish fed with the low-starch groups and inulin suggesting that inulin could induce a stimulation of the host’s metabolism with a low carbohydrate diet, already observed in a 100% plant-based diet [2]. In contrast, no significant differences were observed in the high-starch groups with the inulin intake. This could be explained by the strong effect of the high-starch diets on the microbiota composition and on the expression of many genes, limiting the potential effect of inulin. A significant interaction was measured for the *pk* mRNA expression along with a significant interaction effect on the pyruvate kinase enzymatic activity but not in the same way; indeed, surprisingly, in fish fed low starch, the increase of *pk* mRNAs is associated with a decrease of pyruvate kinase activity. In trout, this gene is already known to be atypically controlled with high-starch diets, with a lower expression of the *pk* gene and a paradoxical increase of pyruvate kinase activity [79–81]. On the other hand, surprisingly also but not for the same reasons, the use of inulin in the high-starch diet resulted in a significant decrease of the glucokinase enzymatic activity, which can be caused by the significant decrease of the *gckb* gene expression.

Interestingly, while the poor glucose homeostasis in rainbow trout fed with fish meal and fish oil (FM/FO) is in part explained by a non-downregulation of the gluconeogenesis pathway with high levels of CHO [79, 82, 83], we did not observed higher expression of *g6pcb2* genes related to gluconeogenesis in the high-starch groups. Additionally, a decrease of glucose-6-phosphatase enzymatic activity was observed with the high-starch diet. These observations suggest the existence of an efficient glucose homeostasis which may explained in part why no post-prandial hyperglycemia was detected in plasma.

Regarding lipid metabolism at the interface with the glucose metabolism, significant interaction CHO/protein × inulin was observed for lipogenesis. In fact, dietary inulin supplementation induced an up-regulation of *serbf1*, *aclyb*, and *aclyc* genes in fish fed the low-starch diets. In fish fed high-starch diets, these genes were down-regulated. Additionally, as previously shown in trout fed plant-based diets [6], the use of a high-starch diet did not result in a higher activity of the fatty acid synthase, with no change in the *fasna* and *fasnb* mRNA expression, while usually the use of a high-starch diet with FM/FO induce an increase of the lipogenesis pathway [77, 83]. Moreover, a decrease of plasma triglycerides was observed with the use of high-starch diets. Finally, no differences in the final body lipid content (Additional file 4: Table S4) were observed, showing that with a 100% plant-based diet the dietary starch in excess does not appear to have been stored as fat (only an expected increase of glycogen was found). In plasma, there was also a significant decrease in cholesterol levels, and it was consistent with the down-regulation of genes related to cholesterol biosynthesis in liver. The lower cholesterol level could be linked to the higher proportion of intestinal LAB, and in particular the presence of *Lactobacillus* species in high-starch diet. Indeed the presence of this bacteria and the production of SCFA have already been linked to a decrease of the cholesterol biosynthesis in human [84].

In carnivorous fish species, the use of plant-based diets, containing for instance pea [85] or soybean protein [86], may induce enteritis of the digestive tract due to the presence of anti-nutritional factors (e.g. non-starch polysaccharides, lectins, tannins) [87]. In salmonids, enteritis can lead to an increase in intestinal epithelial permeability, which can induce an inflammation and leukocyte infiltrations of lamina propria [88]. Moreover, the use of a high CHO diet increased the intestinal permeability and induce inflammation in Chinese perch [89], and largemouth bass [59]. Thus, we studied tight junction proteins (*tjp*) gene expression in mid-intestine. These genes tighten the junctions between epithelial cells to prevent the passage of pathogens through the epithelial barrier, inducing the host’s immune response [90]. In our study, we observed a reduction in *tjp1a* expression in the high-starch diet and down regulation of *tjp3* expression in fish fed with the diets supplemented with inulin. Additionally, two genes coding for tight-junction associated transmembrane proteins, *marveld1* and *marveld3*, were significantly affected by the interaction between starch and inulin, with a reduction of these genes’ expression for HS-0 in comparison with LS-0. The lower expression of these genes in the HS-0 group could be a direct effect of the reduction of antinutritional factors known to cause epithelial damage. Moreover, the use of a high-starch diet did not induce an up-regulation of the *tjp* genes. In addition, the use of inulin in the LS group showed negative effects on intestinal barrier and inflammation-related factors, which is an unexpected result, but the negative effect of inulin on the intestinal barrier has already been observed in gilthead sea bream (*Sparus aurata*) and inulin has not been described as a good immunostimulant [91, 92].

We also studied the effects of the change in the CHO/protein ratio as well as the inulin factors on immunity actors in intestinal mucosa and liver. Indeed, the presence of anti-nutritional factors in the diet can induce innate immune response of epithelial cells and in the liver [93–95]. In this study, a significant decrease of plasma nitric oxide (NO) in fish fed with the high-starch diet was also observed. Interestingly, NO is involved in immune defense in rainbow trout [96], and is particularly linked to the activation of macrophages in site of inflammation in fish [97]. Further studies must be made to assess the inflammatory status of trout, but these results may suggest that the diets formulated with a high CHO/plant protein ratio can have partially reduce the inflammation. Moreover, induction of NO can lead the activation of gene encoding for pro-inflammatory receptors, such as *cxcr4* and *cxcr4.1.1* [96, 97]. In mid intestine, significant interaction between factors was detected for both *cxcr4* and *cxcr4.1.1* gene expression with a lower expression in the HS-0 diet than the LS-0 diet. This result may also be related with the lower proportion of plant protein containing antinutritional factors in the HS-0 group, as antinutritional factors are known to cause enteritis in salmonids [98].

While the use of anti-nutritional factors can lead to mucosal inflammation [94], a high-starch diet in rainbow trout may also result in higher production of liver pro-inflammatory cytokines [95], the liver in teleost being known to be involved in immune responses in teleosts [98, 99]. Interestingly, here, high starch diets induced a significant decrease of the *il1b*, *il8*, and *tnfa* genes, suggesting that reducing of the proportion of plant-proteins may result in a decrease of cytokines responses in the liver. We also observed, surprisingly, that the dietary inclusion of inulin induced an increase in the *il8* expression in the liver. Activation of *il8* may result from tissue damage or infection, which may highlight a potential unexpected adverse effect of dietary inulin.

As an integrative biomarker of immune status in fish, we analyzed the lysozyme known to play a key role in innate immunity by eliminating pathogens [100]: lysozyme is produced and secreted by granulocytes and monocytes during pathogens and parasites infection [101–103]. In our study, we did not observe any difference between the high and low starch groups, but a reduction of this enzymatic activity was observed in the inulin groups. This result with inulin was unexpected since most studies using prebiotics or probiotics in fish have led to an enhancement of the lysozyme activity [25].

In our experiment, the high CHO/protein ratio in a full plant-based diet appears to be beneficial for the health of fish regarding the gut integrity (through tight junction expression genes) and the reduction in the production of pro-inflammatory cytokines in liver. Conversely, inulin reduced lysozyme activity and had no beneficial effect on several immune markers.

The partial replacement of plant proteins by 20% digestible carbohydrates did not reduce growth rates or feed efficiency, while rainbow trout use high levels of protein for growth. This result suggests that in fish fed without marine resources, increasing the CHO/protein ratio could prevent protein catabolism for energy needs (protein sparing effect), whereas trout are often described as poor users of glucose when fed with > 20% of carbohydrates. Although the use of a high carbohydrate/protein ratio did not improve the growth performance, these results support the use of carbohydrates, as these nutrients appear to be properly used by the host, when combined in 100% plant-based diets.

Concerning the reduction of growth performance of trout fed with 2% inulin, several hypotheses can be put forward. Firstly, a significant reduction in feed efficiency (and a trend towards a reduction in daily food intake) was observed in these groups, suggesting that the trout consumed less of the inulin-containing diet, which could explain this reduction in growth. Secondly, the use of 2% inulin in trout fed a 100% plant-based diets may have been too high a proportion, and the lower growth could be explained by an unfavorable inulin/carbohydrate interaction in 100% plant-based diet. Finally, in this experiment we were able to demonstrate that the use of 2% inulin led to an increase in the expression of the gene encoding pro-inflammatory cytokines (IL-8) and a decrease in the activity of lysozyme. This may be a sign of trout’s immune response to inulin consumption and may contribute to reduced growth, but this assumption should be accompanied by further studies.

## Conclusions

To summarize, the inclusion of high-starch diets did not have adverse effects on growth and instead resulted in favorable changes in the intestinal microbiota, short-chain fatty acid (SCFA) production, and glucose metabolism, as previously observed. Additionally, the use of a high amount of starch in the plant-based diet did not elicit an acute inflammatory response. However, the introduction of dietary inulin yielded unexpected outcomes. Inulin had a negative impact on growth, leading to reduced feed efficiency.

### Supplementary Information

**Additional file 1: Table S1.** Mortality count per tanks in fish fed the experimental diets during 12 weeks.

**Additional file 2: Table S2.** Product ions from the reaction of short-chain fatty acids with H_3_O^+^, NO^+^ and O_2_^+^ precursor ions in selected Ion Flow Tube-Mass Spectrometry (from LabSyft software).

**Additional file 3: Table S3.** Primer sequences and used for RT-qPCR analysis.

**Additional file 4: Table S4.** Whole-body composition of rainbow trout fed 100% plant-based diet with high or low levels of dietary carbohydrates and with or without 2% inulin during 12 weeks.

**Additional file 5: Table S5.** Abundances of different phyla in fish fed the experimental diets during 12 weeks.

## Data Availability

All sequence data are available at the NCBI sequence read archive under accession numbers PRJNA953773, https://www.ncbi.nlm.nih.gov/bioproject/PRJNA953773/.

## Abbreviations

bp: Base-pair
CHO: Carbohydrates
Cq: Quantification cycle
DM: Dry matter
ffar: Free fatty acid receptor
FM: Fish meal
FO: Fish Oil
HS: High-starch
HS-0: High-starch with 0 inulin
HS-In: High-starch with 2% inulin
LAB: Lactic-acid bacteria
LS: Low-starch
LS-0: Low-starch with 0 inulin
LS-In: Low-starch with 2% inulin
NO: Nitric oxide
OTU: Operational taxonomic unit
PCoA: Principal correspondence analysis
PCR: Polymerase chain reaction
PER: Protein efficiency ratio
PERMANOVA: Permutational analysis of variance
PLS-DA: Partial least squares discriminant analysis
SCFA: Short-chain fatty acid
SD: Standard deviation
tjp: Tight-junction protein
16S rRNA: 16S ribosomal ribonucleic acid
