# Supplementary material for: Exploring the effects of dietary inulin in rainbow trout fed a high-starch, 100% plant-based diet
Source: J Anim Sci Biotechnol. 2024 Jan 22;15:6. doi: 10.1186/s40104-023-00951-z (PMC10802069; doi:10.1186/s40104-023-00951-z)
Supplement: Supplementary file 1 — Additional file 1: Table S1. Mortality count per tanks in fish fed the experimental diets during 12 weeks. [file 40104_2023_951_MOESM1_ESM.pptx]

## Slide 1
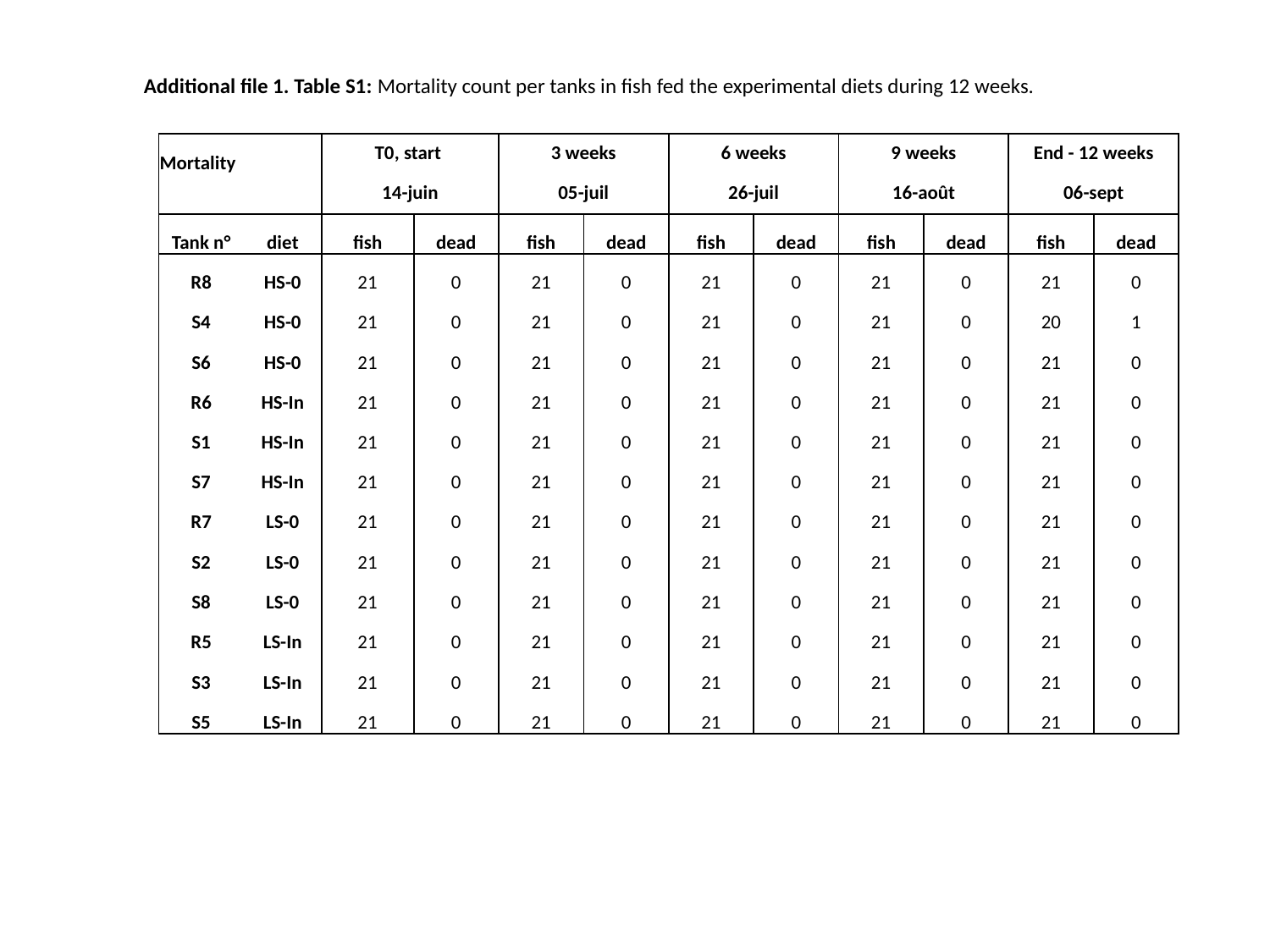

Additional file 1. Table S1: Mortality count per tanks in fish fed the experimental diets during 12 weeks.
| Mortality | | T0, start | | 3 weeks | | 6 weeks | | 9 weeks | | End - 12 weeks | |
| --- | --- | --- | --- | --- | --- | --- | --- | --- | --- | --- | --- |
| | | 14-juin | | 05-juil | | 26-juil | | 16-août | | 06-sept | |
| Tank n° | diet | fish | dead | fish | dead | fish | dead | fish | dead | fish | dead |
| R8 | HS-0 | 21 | 0 | 21 | 0 | 21 | 0 | 21 | 0 | 21 | 0 |
| S4 | HS-0 | 21 | 0 | 21 | 0 | 21 | 0 | 21 | 0 | 20 | 1 |
| S6 | HS-0 | 21 | 0 | 21 | 0 | 21 | 0 | 21 | 0 | 21 | 0 |
| R6 | HS-In | 21 | 0 | 21 | 0 | 21 | 0 | 21 | 0 | 21 | 0 |
| S1 | HS-In | 21 | 0 | 21 | 0 | 21 | 0 | 21 | 0 | 21 | 0 |
| S7 | HS-In | 21 | 0 | 21 | 0 | 21 | 0 | 21 | 0 | 21 | 0 |
| R7 | LS-0 | 21 | 0 | 21 | 0 | 21 | 0 | 21 | 0 | 21 | 0 |
| S2 | LS-0 | 21 | 0 | 21 | 0 | 21 | 0 | 21 | 0 | 21 | 0 |
| S8 | LS-0 | 21 | 0 | 21 | 0 | 21 | 0 | 21 | 0 | 21 | 0 |
| R5 | LS-In | 21 | 0 | 21 | 0 | 21 | 0 | 21 | 0 | 21 | 0 |
| S3 | LS-In | 21 | 0 | 21 | 0 | 21 | 0 | 21 | 0 | 21 | 0 |
| S5 | LS-In | 21 | 0 | 21 | 0 | 21 | 0 | 21 | 0 | 21 | 0 |
