# Supplementary material for: Exploring the effects of dietary inulin in rainbow trout fed a high-starch, 100% plant-based diet
Source: J Anim Sci Biotechnol. 2024 Jan 22;15:6. doi: 10.1186/s40104-023-00951-z (PMC10802069; doi:10.1186/s40104-023-00951-z)
Supplement: Supplementary file 2 — Additional file 2: Table S2. Product ions from the reaction of short-chain fatty acids with H3O+, NO+ and O2+ precursor ions in selected Ion Flow Tube-Mass Spectrometry (from LabSyft software). [file 40104_2023_951_MOESM2_ESM.pptx]

## Slide 1
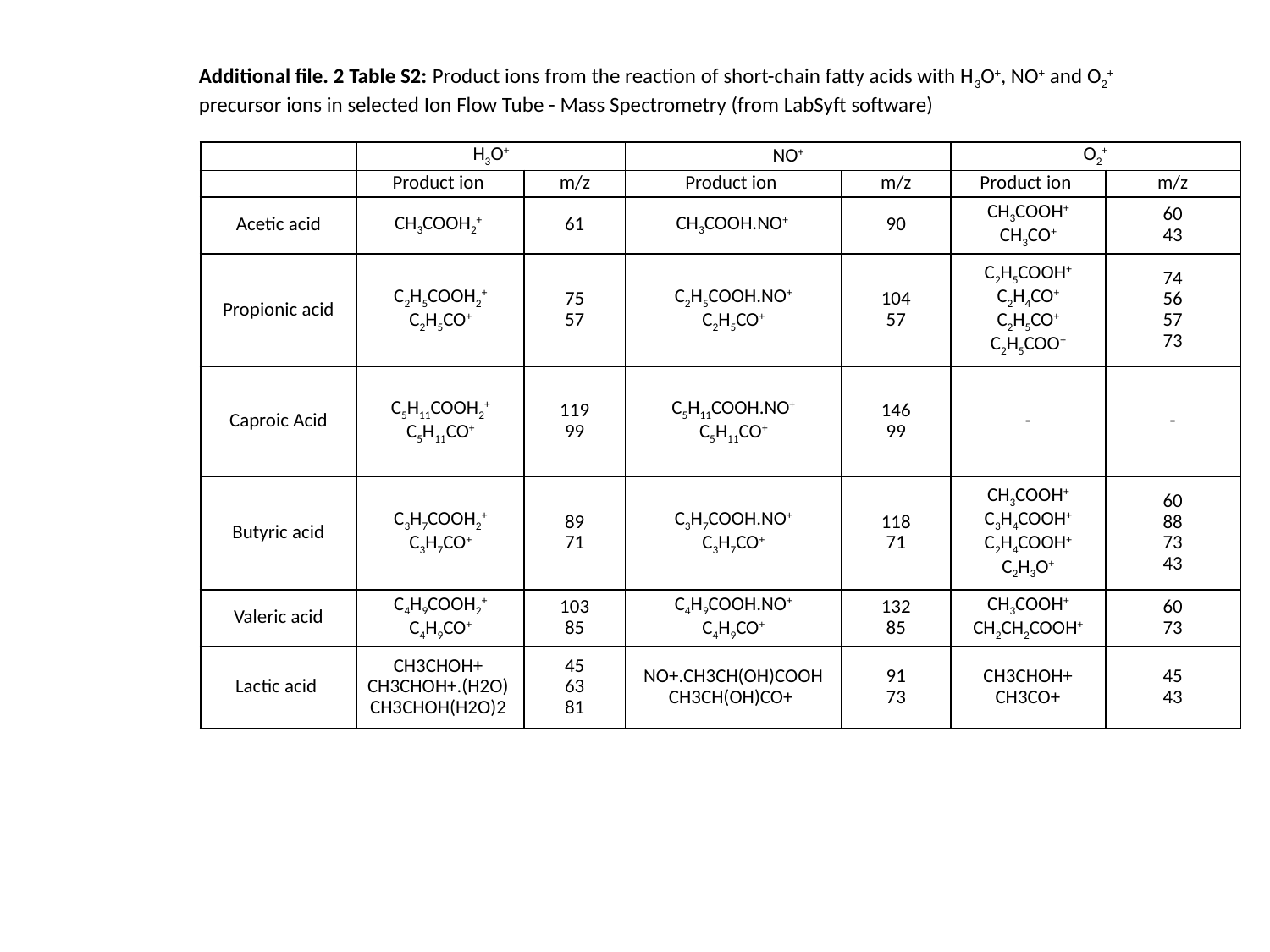

Additional file. 2 Table S2: Product ions from the reaction of short-chain fatty acids with H3O+, NO+ and O2+ precursor ions in selected Ion Flow Tube - Mass Spectrometry (from LabSyft software)
| | H3O+ | | NO+ | | O2+ | |
| --- | --- | --- | --- | --- | --- | --- |
| | Product ion | m/z | Product ion | m/z | Product ion | m/z |
| Acetic acid | CH3COOH2+ | 61 | CH3COOH.NO+ | 90 | CH3COOH+ CH3CO+ | 60 43 |
| Propionic acid | C2H5COOH2+ C2H5CO+ | 75 57 | C2H5COOH.NO+ C2H5CO+ | 104 57 | C2H5COOH+ C2H4CO+ C2H5CO+ C2H5COO+ | 74 56 57 73 |
| Caproic Acid | C5H11COOH2+ C5H11CO+ | 119 99 | C5H11COOH.NO+ C5H11CO+ | 146 99 | - | - |
| Butyric acid | C3H7COOH2+ C3H7CO+ | 89 71 | C3H7COOH.NO+ C3H7CO+ | 118 71 | CH3COOH+ C3H4COOH+ C2H4COOH+ C2H3O+ | 60 88 73 43 |
| Valeric acid | C4H9COOH2+ C4H9CO+ | 103 85 | C4H9COOH.NO+ C4H9CO+ | 132 85 | CH3COOH+ CH2CH2COOH+ | 60 73 |
| Lactic acid | CH3CHOH+ CH3CHOH+.(H2O) CH3CHOH(H2O)2 | 45 63 81 | NO+.CH3CH(OH)COOH CH3CH(OH)CO+ | 91 73 | CH3CHOH+ CH3CO+ | 45 43 |
