# Supplementary material for: Exploring the effects of dietary inulin in rainbow trout fed a high-starch, 100% plant-based diet
Source: J Anim Sci Biotechnol. 2024 Jan 22;15:6. doi: 10.1186/s40104-023-00951-z (PMC10802069; doi:10.1186/s40104-023-00951-z)
Supplement: Supplementary file 3 — Additional file 3: Table S3. Primer sequences and used for RT-qPCR analysis. [file 40104_2023_951_MOESM3_ESM.pptx]

## Slide 1
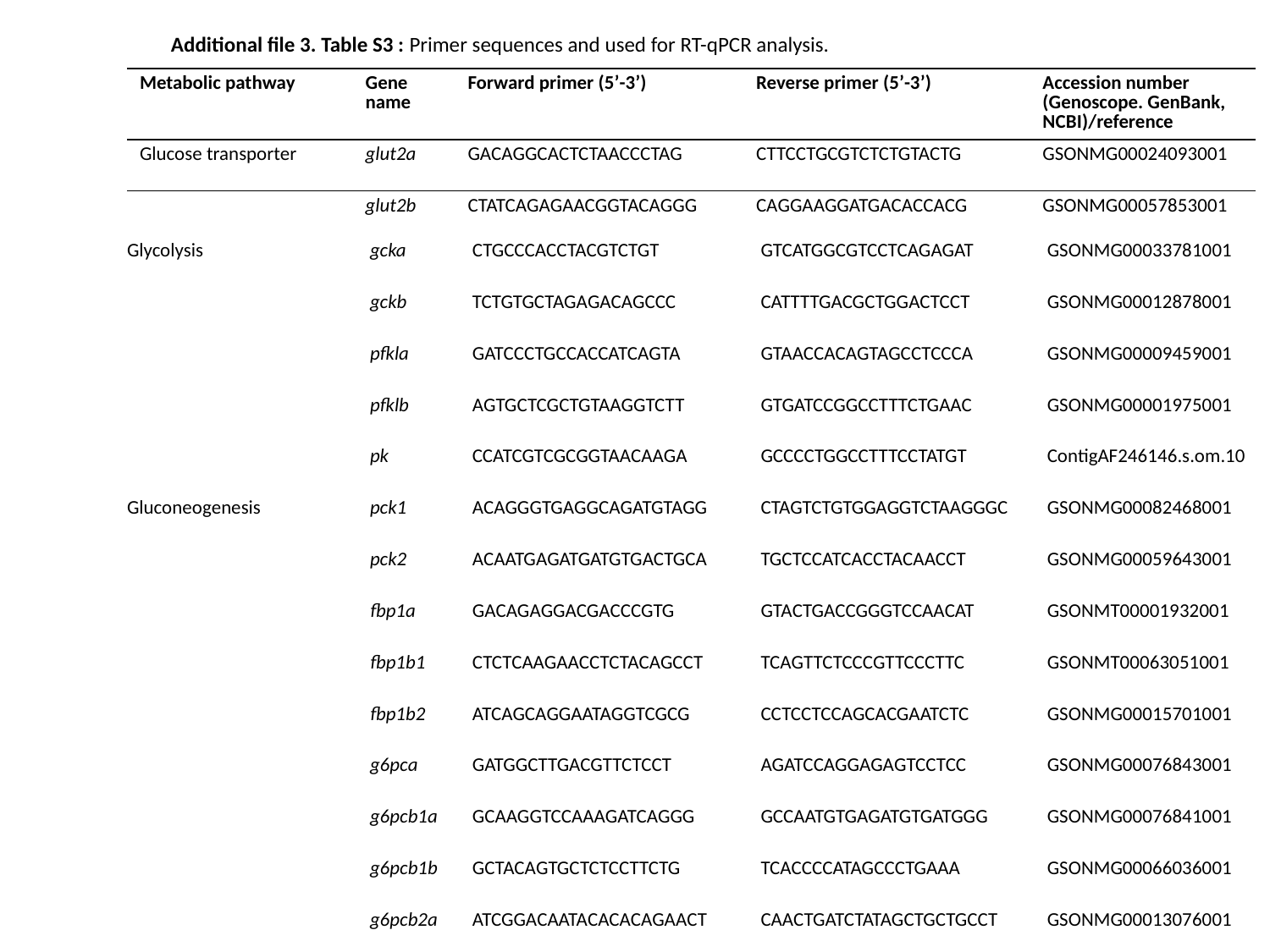

Additional file 3. Table S3 : Primer sequences and used for RT-qPCR analysis.
| Metabolic pathway | Gene name | Forward primer (5’-3’) | Reverse primer (5’-3’) | Accession number (Genoscope. GenBank, NCBI)/reference |
| --- | --- | --- | --- | --- |
| Glucose transporter | glut2a | GACAGGCACTCTAACCCTAG | CTTCCTGCGTCTCTGTACTG | GSONMG00024093001 |
| | glut2b | CTATCAGAGAACGGTACAGGG | CAGGAAGGATGACACCACG | GSONMG00057853001 |
| Glycolysis | gcka | CTGCCCACCTACGTCTGT | GTCATGGCGTCCTCAGAGAT | GSONMG00033781001 |
| | gckb | TCTGTGCTAGAGACAGCCC | CATTTTGACGCTGGACTCCT | GSONMG00012878001 |
| | pfkla | GATCCCTGCCACCATCAGTA | GTAACCACAGTAGCCTCCCA | GSONMG00009459001 |
| | pfklb | AGTGCTCGCTGTAAGGTCTT | GTGATCCGGCCTTTCTGAAC | GSONMG00001975001 |
| | pk | CCATCGTCGCGGTAACAAGA | GCCCCTGGCCTTTCCTATGT | ContigAF246146.s.om.10 |
| Gluconeogenesis | pck1 | ACAGGGTGAGGCAGATGTAGG | CTAGTCTGTGGAGGTCTAAGGGC | GSONMG00082468001 |
| | pck2 | ACAATGAGATGATGTGACTGCA | TGCTCCATCACCTACAACCT | GSONMG00059643001 |
| | fbp1a | GACAGAGGACGACCCGTG | GTACTGACCGGGTCCAACAT | GSONMT00001932001 |
| | fbp1b1 | CTCTCAAGAACCTCTACAGCCT | TCAGTTCTCCCGTTCCCTTC | GSONMT00063051001 |
| | fbp1b2 | ATCAGCAGGAATAGGTCGCG | CCTCCTCCAGCACGAATCTC | GSONMG00015701001 |
| | g6pca | GATGGCTTGACGTTCTCCT | AGATCCAGGAGAGTCCTCC | GSONMG00076843001 |
| | g6pcb1a | GCAAGGTCCAAAGATCAGGG | GCCAATGTGAGATGTGATGGG | GSONMG00076841001 |
| | g6pcb1b | GCTACAGTGCTCTCCTTCTG | TCACCCCATAGCCCTGAAA | GSONMG00066036001 |
| | g6pcb2a | ATCGGACAATACACACAGAACT | CAACTGATCTATAGCTGCTGCCT | GSONMG00013076001 |
| | g6pcb2b | CCTCTGCTCTTCTGACGTAG | TGTCCATGGCTGCTCTCTAG | GSONMG00014864001 |
| Lipogenesis de novo | srebf1a | CAGTTGCTGCTGTGTGACCT | TGATGTGTTCGTGTGGGACT | XM\_021624594.1 |
| | g6pdb | TGTCACAGGGTCAACATAATGC | AGCAGCAACATTGTGACAAGA | XM\_021566624.1 |
| | aclya | GGTGTGAGCGTTATGAAGCA | GATTTCAGCCGGCAATTCTA | XM\_021624819.1 |
| | aclyb | GACGGAAAAGTCCTGATCATC | TGTGAGTCTCTGTGCCGAAG | XM\_021557697.1 |
| | aclyc | ACCGGTTCACAAATGGGATA | CCAAGTCACCCAGAAAGCAT | XM\_021575392.1 |
| | aca-aa | ACAGGACCCTAAAGCACAGG | GGTGAAAGAGGTGTCCAGGA | XM\_021623125.1 |
| | aca-ab | TCCAGTTCATGCTGCCTACC | GCTTAATGTCCCGAGTGCGA | XM\_021618451.1 |
| | aca-ba | TCGCTCAGAATTCCGGGTAC | CGCGTGGTGATGGTTACAAT | XM\_021605386.1 |
| | aca-bb | TGAACAGCTTGGTAAACAGCC | TCTCGTGCATTCTACCAGGG | XM\_021620987.1 |
| | fasna | AGCTCGTACCTTCCTGTTCC | ACAACAAGGCCGTTCTTCAG | XM\_021576228.1 |
| | fasnb | CTGCTACAACTGGAAACACGC | ATCAAGGTAACGACACACCGT | XM\_021581290.1 |
| Fatty acids β-oxidation | hadh | TTATCATGGACGGCTGGGTT | CAGCTCGTGTTGATTCTGGG | XM\_021573383.1 |
| | cpt1a | CGTTAGCAATGCCTCACAGA | AACGGAACAGGTAGCGCTAT | XM\_021607999.1 |
| | cpt1ba | AGCAAGCTTGAATTGAATCCCT | CTTATGGCGCGCTTTATCCA | NM\_001171855.1 |
| | cpt1bb | GGTGTCCAGTTGCAGAAAGG | TGACTTTGGTTTCTCACAGTGG | NM\_001124735.1 |
| | acox1a | ACATCGTTTCATAGACTGCCAG | CTCAATCCTTTATCCTCCATCCA | XM\_021568071.1 |
| | acox3 | ACCTCCTCAGCCAACTACAC | CCTTAGCCACTCTCCTCTCG | XM\_021577711.1 |
| Cholesterol metabolism | srebp2a | AGACCCACACACGTTAATGCT | CTATGTGACTGTTCTGGGATC | XM\_021558053.1 |
| | srebp2b | AATAGTCAACAACGCGAACAGT | GGTTGGCAGTGTGAAGATTGA | XM\_021625098.1 |
| | hmgcra | GGAGGTGATCGTAGGCACTG | GGCTATGCACCGTGTGATGG | XM\_021622177.1 |
| | hmgcrb | GTGTTGTTTCGGCTGCATGT | GCCATAACACGGGCAAAGTG | XM\_021602157.1 |
| | cyp51a | TAAGAGACACTGAGCGCAAACA | TTATGAGAGCCAGCCCTATGAG | XM\_021560395.1 |
| | cyp51b | CCTGGTCGATGGCTACTTCC | TGCTCTTCTGGGTGTAACCT | XM\_021572174.1 |
| | dhcr7a | ACTGCCAGCTTTGTGCTCTA | ACCCATGGCAACTAACTGCT | XM\_021585986.1 |
| | dhcr7b | AGTTGGCGTTGGTCAAGTAAC | TTCATGTAACGCTGGGCTGA | XM\_021607019.1 |
| | lxraa | AGCAAGTGTGTACTCGTCAGA | GAAACGAACCCTTCTGCTTGTC | NM\_001159338 |
| | lxrab | CAGAGTCAGGACCCCCAGAA | GTTGAGGTCTTCAGCAGCGC | XM\_021585769.1 |
| | abca1a | AACGAGGTTGGAGGTATCAATG | ACCGAATCTTTCCAGAGCGT | XM\_021584015.1 |
| | abca1b | GTCCATACCGGAGGTCCCAG | CAGGGAGCATTTTCTTGCGGC | XM\_021561291.1 |
| | abcg4b | AATCGTTCGGAGCTGGAGTG | GCATCCTCCACGTCCAGTAG | XM\_021619008.1 |
| Pro-inflammatory cytokines and chemokines receptors | il1b | ACGGTTCGCTTCCTCTTCTACA | GCTCCAGTGAGGTGCTGATGAA | AJ245925.2 |
| | il8 | GTCAGCCAGCCTTGTCGTTGT | CGTCTGCTTTCCGTCTCAATGC | NM\_001124362.1 |
| | tnfa | GGCGAGCATACCACTCCTCTGA | AGCTGGAACACTGCACCAAGGT | NM\_001124362.1 |
| | cxcr4 | TCAAGAGTTCTGCTCGCCG | TCTGACTCTGTCGTTGTGGAC | XM\_021594980.2 |
| | cxcr4.1.1 | GTTCCGTTTCCAGCACATTCT | ATGATGCAGTAGCAGGTGAGG | NM\_001124342.2 |
| Tight junction proteins | tjp1a | TCAGCTGCGCTATGATGAGG | GGTCGTAACGTAGAGGAGCG | XM\_036980662.1 |
| | tjp3 | GGGACACAGCCGTGATAGTT | TGACACGCCATTGACCATGA | XM\_031822609.1 |
| | marveld1 | GGTGATCGGCGTCATGATCT | GTGGCAGCCAGGTAGACTTT | XM\_021576418.2 |
| | marveld3 | TTCCACCAGCTCAAGCTACC | GGGACTCTCAAAACCCCGAA | XM\_021586195.2 |
| FFARs | ffar1 | ACT GTT GCA CCT GAG TCT GG | GCT GGT CCT GGG TGA AGT TC | ENSOMYG00000041396 |
| | ffar2a1a | CCG AGT TCC TCT GCT CCA TC | TAG GTG ATG GGG AAG GCA AC | ENSOMYG00000004986 |
| | ffar2a2 | GAC AAC TTC ACC CAG GAG CA | AGC AGA ACA CAC AGG CCA G | ENSOMYG00000030315 |
| | ffar2b1.1 | TTT TCC ACA CAC AGT TGG CC | AGG TAG TGT TGT CGG CAT CT | ENSOMYG00000041393 |
| | ffar2b1.2 | GTG TGG CCT TCC CTA TCA GA | GCA GGG CAC AAT GTA CAC AA | ENSOMYG00000041387 |
| | ffar2b2a | CCC ATC CAA CAC TCG CTG AA | TGA TGA CGA CGA TGC TCA GG | ENSOMYG00000030493 |
| | ffar2b2b1 | TGA CCG CAA TCA GTG TCG AA | CCC AGA AGA AGA CGC TAG CC | ENSOMYG00000030500 |
| | ffar2b2b2 | GTC CAG TAC CAT CAA CGC CA | CTG CAC ACT CTC CAA CAG GGT | ENSOMYG00000005604 |
| House-keeping genes | eef1a | TCCTCTTGGTCGTTTCGCTG | ACCCGAGGGACATCCTGTG | XM\_021567853.1 |
| | b actin | GATGGGCCAGAAAGACAGCTA | TCGTCCCAGTTGGTGACGAT | - |
