# Supplementary material for: Exploring the effects of dietary inulin in rainbow trout fed a high-starch, 100% plant-based diet
Source: J Anim Sci Biotechnol. 2024 Jan 22;15:6. doi: 10.1186/s40104-023-00951-z (PMC10802069; doi:10.1186/s40104-023-00951-z)
Supplement: Supplementary file 4 — Additional file 4: Table S4. Whole-body composition of rainbow trout fed 100% plant-based diet with high or low levels of dietary carbohydrates and with or without 2% inulin during 12 weeks. [file 40104_2023_951_MOESM4_ESM.pptx]

## Slide 1
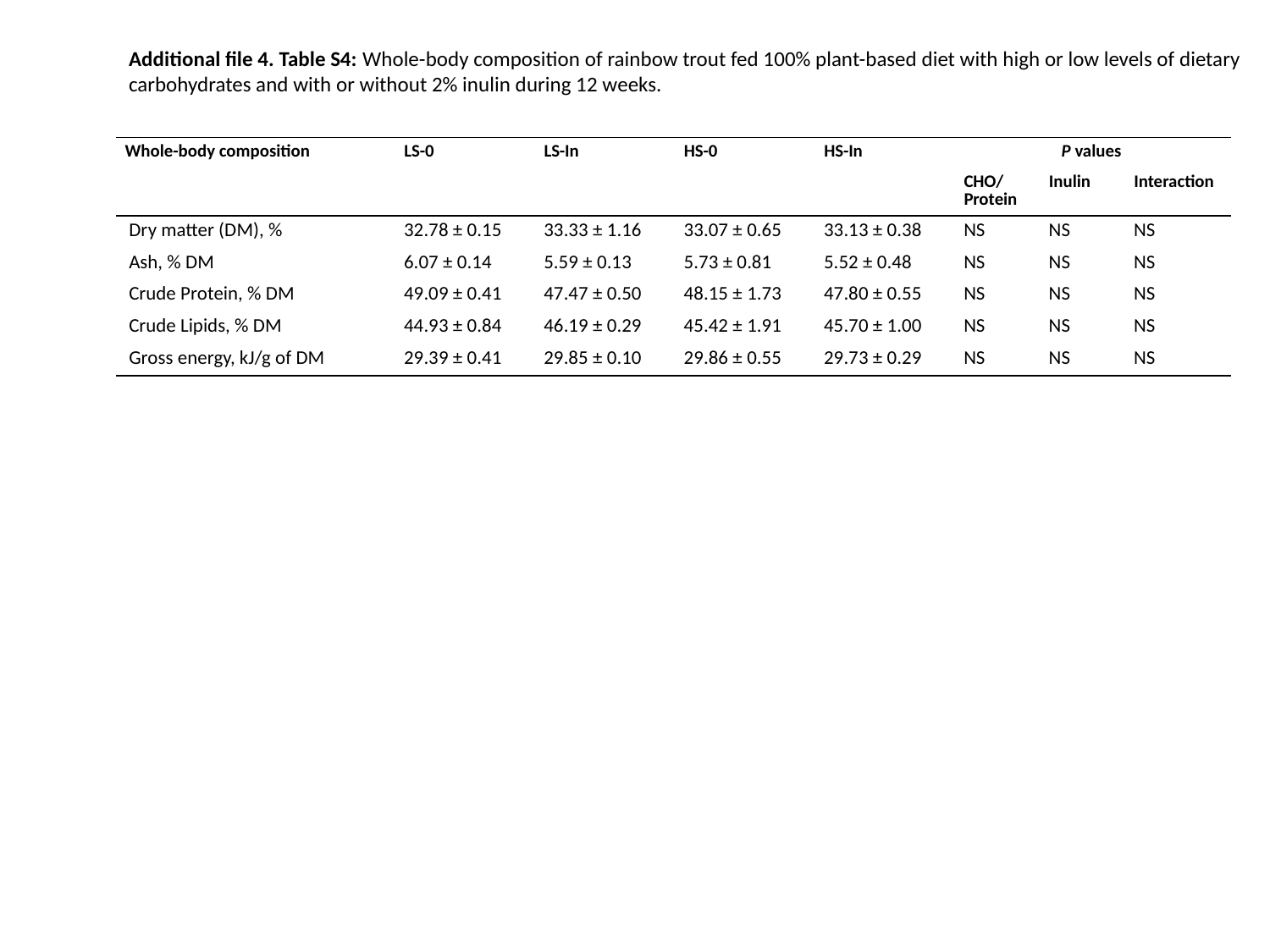

Additional file 4. Table S4: Whole-body composition of rainbow trout fed 100% plant-based diet with high or low levels of dietary carbohydrates and with or without 2% inulin during 12 weeks.
| Whole-body composition | LS-0 | LS-In | HS-0 | HS-In | P values | | |
| --- | --- | --- | --- | --- | --- | --- | --- |
| | | | | | CHO/ Protein | Inulin | Interaction |
| Dry matter (DM), % | 32.78 ± 0.15 | 33.33 ± 1.16 | 33.07 ± 0.65 | 33.13 ± 0.38 | NS | NS | NS |
| Ash, % DM | 6.07 ± 0.14 | 5.59 ± 0.13 | 5.73 ± 0.81 | 5.52 ± 0.48 | NS | NS | NS |
| Crude Protein, % DM | 49.09 ± 0.41 | 47.47 ± 0.50 | 48.15 ± 1.73 | 47.80 ± 0.55 | NS | NS | NS |
| Crude Lipids, % DM | 44.93 ± 0.84 | 46.19 ± 0.29 | 45.42 ± 1.91 | 45.70 ± 1.00 | NS | NS | NS |
| Gross energy, kJ/g of DM | 29.39 ± 0.41 | 29.85 ± 0.10 | 29.86 ± 0.55 | 29.73 ± 0.29 | NS | NS | NS |
