# Supplementary material for: Exploring the effects of dietary inulin in rainbow trout fed a high-starch, 100% plant-based diet
Source: J Anim Sci Biotechnol. 2024 Jan 22;15:6. doi: 10.1186/s40104-023-00951-z (PMC10802069; doi:10.1186/s40104-023-00951-z)
Supplement: Supplementary file 5 — Additional file 5: Table S5. Abundances of different phyla in fish fed the experimental diets during 12 weeks. [file 40104_2023_951_MOESM5_ESM.pptx]

## Slide 1
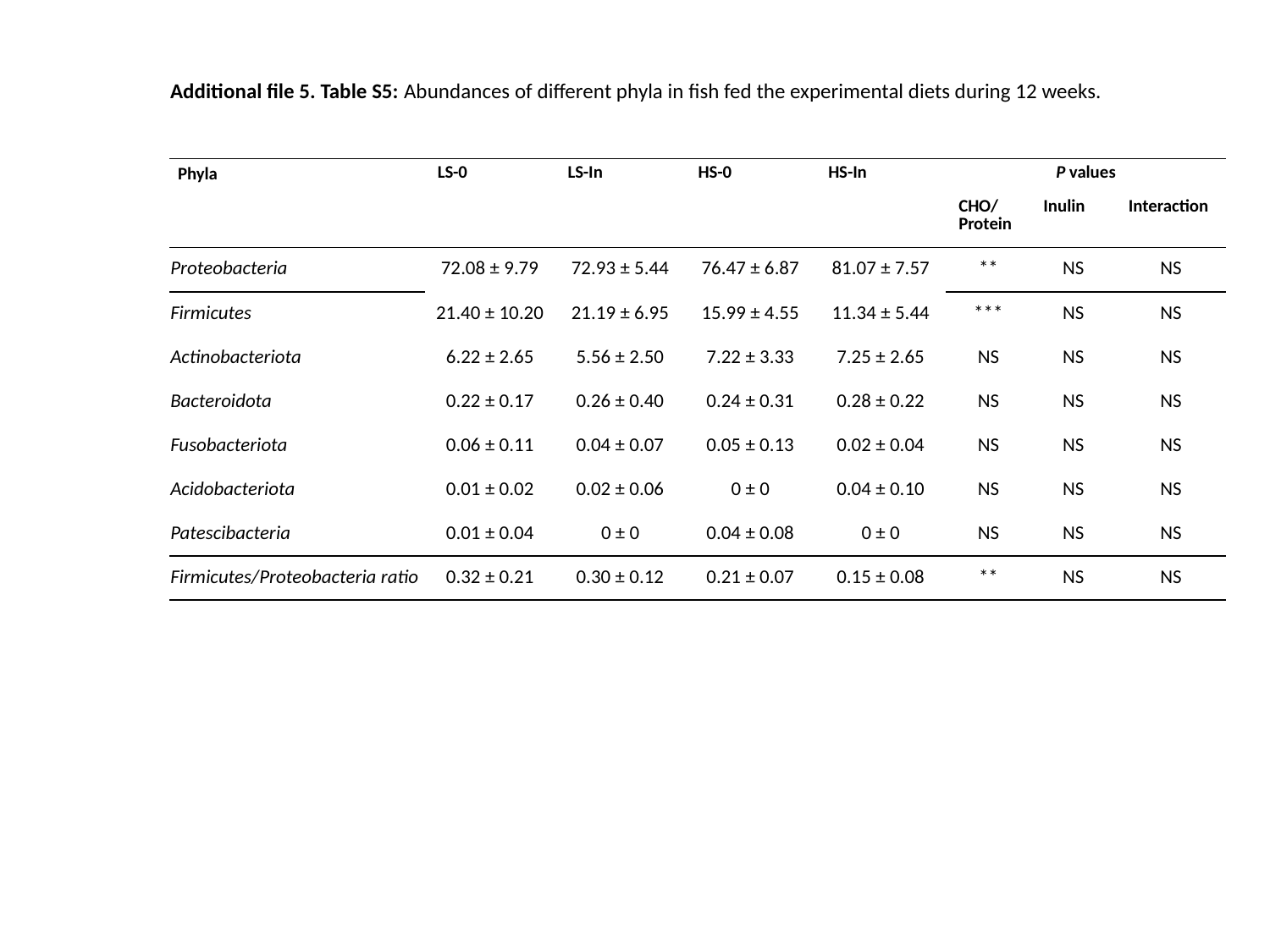

Additional file 5. Table S5: Abundances of different phyla in fish fed the experimental diets during 12 weeks.
| Phyla | LS-0 | LS-In | HS-0 | HS-In | P values | | |
| --- | --- | --- | --- | --- | --- | --- | --- |
| | | | | | CHO/ Protein | Inulin | Interaction |
| Proteobacteria | 72.08 ± 9.79 | 72.93 ± 5.44 | 76.47 ± 6.87 | 81.07 ± 7.57 | \*\* | NS | NS |
| Firmicutes | 21.40 ± 10.20 | 21.19 ± 6.95 | 15.99 ± 4.55 | 11.34 ± 5.44 | \*\*\* | NS | NS |
| Actinobacteriota | 6.22 ± 2.65 | 5.56 ± 2.50 | 7.22 ± 3.33 | 7.25 ± 2.65 | NS | NS | NS |
| Bacteroidota | 0.22 ± 0.17 | 0.26 ± 0.40 | 0.24 ± 0.31 | 0.28 ± 0.22 | NS | NS | NS |
| Fusobacteriota | 0.06 ± 0.11 | 0.04 ± 0.07 | 0.05 ± 0.13 | 0.02 ± 0.04 | NS | NS | NS |
| Acidobacteriota | 0.01 ± 0.02 | 0.02 ± 0.06 | 0 ± 0 | 0.04 ± 0.10 | NS | NS | NS |
| Patescibacteria | 0.01 ± 0.04 | 0 ± 0 | 0.04 ± 0.08 | 0 ± 0 | NS | NS | NS |
| Firmicutes/Proteobacteria ratio | 0.32 ± 0.21 | 0.30 ± 0.12 | 0.21 ± 0.07 | 0.15 ± 0.08 | \*\* | NS | NS |
